# Renal sarcomas in children and adolescents: a retrospective, multicenter cohort study

**DOI:** 10.1016/j.eclinm.2025.103713

**Published:** 2025-12-18

**Authors:** Katlyn G. McKay, Catherine Beckhorn, Nelly-Ange T. Kontchou, Zachary J. Kastenberg, Jonathan Roach, Bhargava Mullapudi, Timothy B. Lautz, Roshni Dasgupta, Lindsay J. Talbot, Jennifer H. Aldrink, Nelson Piché, Brian T. Craig, Barrett Cromeens, Shannon L. Castle, Joshua Short, Robin T. Petroze, Peter Mattei, David H. Rothstein, Elizabeth A. Fialkowski, Barrie S. Rich, Erin G. Brown, Natashia M. Seemann, Hau D. Le, Tamer M. Ahmed, Erika A. Newman, Christa N. Grant, Stephanie F. Polites, Danielle B. Cameron, Eugene S. Kim, Mary T. Austin, Brian A. Coakley, Joseph T. Murphy, Chloé Boehmer, Marcus M. Malek, Elisabeth Tracy, Harold N. Lovvorn, Pediatric Surgical Oncology Research Collaborativeaj, Heidi Chen, Harold J. Leraas, David W. Hoyt, Emily K. Myers, Nicolas G. Cost, Charles R. Marchese, Amanda R. Jensen, Michela M. Carter, John Lundstedt, Joseph G. Brungardt, Andrew M. Davidoff, Andrew J. Murphy, Sara Mansfield, Dave R. Lal, Jennifer M. Schuh, Sindhu V. Mannava, Adriana Lopez, Kelsey Mello, Shay Rajaval, Grace R. Thompson, Kathryn L. Fowler, Nathan Martchenke, Richard D. Glick, Kathleen Doyle, Paige Abril, Hannah N. Rinehardt, Jacob Davidson, Claire A. Wilson, Devashish Joshi, Michael Stellon, Alexandra Dimmer, Keyonna Williams, Maya Hammoud, Merit Gorgy, Julia G. Debertin, Alyssa Stetson, William G. Lee, Aaron Barkhordar, Lauren K. Mayon, Anastasia Kahan, Michael Pitonak

**Affiliations:** a Department of Surgery, Vanderbilt University Medical Center, Nashville, TN, USA; b Duke University School of Medicine, Durham, NC, USA; c Department of Surgery, Division of Pediatric Surgery, University of Utah, Primary Children's Hospital, Salt Lake City, UT, USA; d Department of Pediatric Surgery, Children's Hospital of Colorado, Denver, CO, USA; e Children's Mercy Hospital, Kansas City, MO, USA; f Department of Surgery, Division of Pediatric Surgery, Lurie Children's Hospital, Northwestern School of Medicine, Chicago, IL, USA; g Division of Pediatric Surgery, Cincinnati Children's Medical Center, Cincinnati, OH, USA; h Department of Surgery, St. Jude Children's Research Hospital, Memphis, TN, USA; i Division of Pediatric Surgery, Department of Surgery, Nationwide Children's Hospital, The Ohio State University College of Medicine, Columbus, OH, USA; j Division of Pediatric Surgery, Centre Hospitalier Universitaire Ste-Justine, Université de Montréal, Montréal, Quebec, Canada; k Division of Pediatric Surgery, Medical College of Wisconsin, Children's Wisconsin, Milwaukee, WI, USA; l Division of Pediatric Surgery, Riley Hospital for Children, Indiana University School of Medicine, Indianapolis, IN, USA; m Division of Pediatric Surgery, Valley Children's Hospital, Madera, CA, USA; n Pediatric Surgical Associates, Children's Minnesota, Minneapolis, MN, USA; o Division of Pediatric Surgery, C.S. Mott Children's Hospital, University of Michigan, Ann Arbor, MI, USA; p General, Thoracic and Fetal Surgery, Children's Hospital of Philadelphia, Philadelphia, PA, USA; q Division of Pediatric General and Thoracic Surgery, University of Washington, Seattle, WA, USA; r Department of Surgery, Oregon Health & Science University, Portland, OR, USA; s Division of Pediatric Surgery, Feinstein/Northwell, Cohen Children's Medical Center, Queens, NY, USA; t Division of Pediatric Surgery, Department of Surgery, University of California Davis Children's Hospital, Sacramento, CA, USA; u Division of Pediatric Surgery, Children's Hospital, London Health Sciences Centre, London, ON, Canada; v Division of Pediatric Surgery, American Family Children's Hospital, University of Wisconsin School of Medicine and Public Health, Madison, WI, USA; w Division of Pediatric Surgery, SUNY Upstate Medical University, Syracuse, NY, USA; x Division of Pediatric Surgery, Westchester Medical Center, New York Medical College, Valhalla, NY, USA; y Department of Surgery, Mayo Clinic, Rochester, MN, USA; z Massachusetts General Hospital, Boston, MA, USA; aa Division of Pediatric Surgery, Cedars-Sinai Medical Center, Los Angeles, CA, USA; ab Division of Pediatric Surgery, University of Texas MD Anderson Cancer Center, Houston, TX, USA; ac Division of Pediatric Surgery, Department of Surgery, Icahn School of Medicine Mount Sinai, New York, NY, USA; ad Division of Pediatric Surgery, Children's Health Children's Medical Center, University of Texas Southwestern, Dallas, TX, USA; ae Division of Pediatric General and Thoracic Surgery, UPMC Children's Hospital of Pittsburgh, Pittsburgh, PA, USA; af Department of Pediatric Surgery, Duke University Medical Center, Durham, NC, USA; ag Department of Pediatric Surgery, Vanderbilt University Medical Center, Nashville, TN, USA

**Keywords:** Renal sarcoma, Clear cell sarcoma of the kidney, Ewing sarcoma, Anaplastic sarcoma of the kidney, DICER1, Children

## Abstract

**Background:**

Renal sarcomas arise rarely in children and adolescents and represent a histologically and biologically diverse disease category. Consequently, standardizing optimal therapies for pediatric renal sarcomas remains challenging. Leveraging a large North American research collaborative, the purposes of this study were to evaluate the current state of patient, disease, and survival characteristics among pediatric renal sarcomas and to expose knowledge gaps that will inform future discovery.

**Methods:**

Patients 21 years or younger and treated for a primary renal sarcoma between January 1st, 2000 and November 30th, 2022 were identified through the Pediatric Surgical Oncology Research Collaborative. Patient (e.g., demographics) and disease (e.g., histology, stage, molecular alterations) characteristics were abstracted from contributing institutions. Descriptive statistics, Pearson-Chi square (categorical variables), Kruskal–Wallis (continuous variables), Cox regression (Hazard ratios), and Kaplan–Meier 4-year event-free and overall survival (OS) analyses were completed.

**Findings:**

Among 158 patients, clear cell sarcoma of the kidney (CCSK; n = 94), Ewing sarcoma (EWS; n = 33), and undifferentiated sarcoma (n = 8) predominated. Sarcoma type correlated significantly with age at diagnosis (p < 0.0001), with infantile fibrosarcoma (IFS) and CCSK occurring in the youngest patients, whereas EWS and synovial sarcoma presented in the oldest. Predisposition syndromes were identified in 11/155 (7.1%) patients, most commonly DICER1 and Li-Fraumeni. Multimodal therapies varied significantly across sarcoma types (p = 0.0008), although nephrectomy was uniform. Tumor thrombectomy was performed in 9 patients (6 with EWS). When tested, somatic molecular alterations were observed principally in CCSK (17/38; 45%) and EWS (26/26; 100%; p = 0.001). At 4 years, OS differed significantly by sarcoma type, ranging from highest to lowest as follows: CCSK 0.927 (95% CI 0.845–0.967), EWS 0.901 (95% CI 0.723–0.967), undifferentiated sarcoma 0.833 (95% CI 0.273–0.975), IFS 0.667 (95% CI 0.054–0.945), and rhabdomyosarcoma 0.500 (95% CI 0.111–0.804; p = 0.036). Hematogenous metastases occurred most in the lungs (n = 19 total; 10 with EWS), followed by bone (n = 12), which occurred only with CCSK (n = 9) and EWS (n = 3). Two patients developed brain metastases (one each with CCSK and rhabdomyosarcoma). At 4 years, OS was 0.957 (95% CI 0.888–0.984) for patients presenting without metastases and 0.717 (95% CI 0.545–0.833) for those with metastases (p = 0.00015).

**Interpretation:**

Renal sarcomas presenting in children and adolescents comprise a heterogeneous disease category with unique patient, clinical, and molecular characteristics that complicate standardizing therapeutic strategies beyond CCSK and EWS.

**Funding:**

None.

**Research in context**

**Evidence before this study**

Primary renal sarcomas in children and adolescents are rare and heterogeneous in histology and biology, which has hindered precise risk stratification and standardization of therapy. The PubMed database was searched from inception to April 2025 without language restrictions using combinations of terms including “renal sarcoma,” “children,” “pediatric,” “adolescent,” “clear cell sarcoma of the kidney,” “Ewing sarcoma AND kidney,” “anaplastic sarcoma of the kidney,” “infantile fibrosarcoma,” “synovial sarcoma,” and “rhabdomyosarcoma.” Reference lists of relevant reports were hand-searched to ensure comprehensive capture. For most pediatric renal sarcoma types, the available literature consisted of single-institution case reports and small retrospective series, with very limited prospective data. An important exception was clear cell sarcoma of the kidney (CCSK), for which a recent Children's Oncology Group (COG) effort demonstrated the value of centralized risk assignment and standardized, multi-institutional care to achieve excellent survival. However, multiple other sarcoma types that arise in the pediatric kidney are even rarer than CCSK, remain poorly characterized biologically, and lack consensus therapeutic strategies derived from cooperative trials. Key gaps include histology-specific natural history, metastatic patterns, late health effects, and the role of germline and somatic genomics in guiding therapy.

**Added value of this study**

Leveraging the North American Pediatric Surgical Oncology Research Collaborative, a multi-institutional cohort was assembled of 158 patients aged 21 years or younger and treated for primary renal sarcomas between 2000 and 2022. Standardized data abstraction enabled harmonized analyses of demographics, histology, stage, metastatic patterns, molecular alterations, treatment, late effects, and survival. While our CCSK cohort complements recent COG findings, this study reports, to our knowledge, the largest series of renal Ewing sarcoma (EWS) in children and adolescents (n = 33) and provides detailed characterization across nine additional rare histologies. This study showed that sarcoma type strongly correlates with age at diagnosis, with infantile fibrosarcoma and CCSK presenting in the youngest patients, and EWS and synovial sarcoma in the oldest. Predisposition syndromes were identified in 7.1% of patients (DICER1 and Li-Fraumeni most common). Therapeutic approaches varied by histology, but nephrectomy was nearly universal; inferior vena cava thrombectomy was required in selected cases and notably for EWS. Somatic alterations were frequent in CCSK (45% of those tested) and universal in EWS (100% of those tested). Four-year overall survival differed by sarcoma type—CCSK 92.6%, EWS 90.1%, undifferentiated 83.3%, infantile fibrosarcoma 67.7%, rhabdomyosarcoma 50%—and was significantly lower with metastatic presentation (71.7% vs 95.7%). Distinct dissemination patterns emerged: lungs were the most common metastatic site overall, whereas bone involvement was confined to CCSK and EWS; brain metastases were rare. Importantly, assessment of late effects identified a specific risk of chronic renal insufficiency among survivors of renal EWS.

**Implications of all the available evidence:**

Advances in histologic and molecular classification have revealed that pediatric renal sarcomas encompass biologically distinct malignancies historically grouped as unfavorable histology Wilms tumor. These findings reinforce that histology, stage, and metastatic pattern are major determinants of outcome and that different sarcoma types follow unique routes of spread that challenge cure. Together with prior cooperative data in CCSK, these results argue for histology- and biology-driven, internationally coordinated trials; routine, high-quality molecular profiling to refine diagnosis and risk; and harmonized treatment algorithms tailored to sarcoma type. Surveillance strategies should reflect dissemination patterns (for example, targeted bone evaluation in CCSK and EWS), and survivorship care should proactively monitor renal function, particularly after EWS-directed therapy. Given the rarity of these tumors, sustained global collaboration, standardized data elements, and longitudinal capture of late effects are essential to close knowledge gaps and to enable development of cell-specific therapies that can further improve survival while minimizing long-term toxicity.

## Introduction

Renal sarcomas arise rarely in children and adolescents, comprising an estimated 6% of all pediatric kidney cancers.^1^, ^2^, ^3^, ^4^ Clear cell sarcoma of the kidney (CCSK) predominates this unusual and scarce category of malignant pediatric mesenchymal tumors. A current report from the Children's Oncology Group (COG) documented 4-year event-free (EFS) and overall (OS) survival from CCSK at 85.3% and 94.6%, respectively, following treatment optimization on high-risk protocol, AREN0321, or its umbrella study, AREN03B2.^5^ Given these excellent survival results, the recent focus of the COG has been on tailoring therapeutic intensity to meet the specific disease burden of CCSK. Other international pediatric oncology cooperative groups, principally the International Society of Pediatric Oncology (SIOP) and Japan Children's Cancer Group, through standardizing therapies for CCSK have also achieved excellent survival rates for this challenging pediatric renal cancer.^6^^,^^7^ While somatic molecular events have been revealed in CCSK recently, their collective impact on treatment resistance is insufficiently characterized to date, given the relative infrequency of this malignancy, and so, precise risk-assignment according to tumor biology remains a keen priority for future studies of COG and SIOP.^2^^,^^8^, ^9^, ^10^, ^11^

But multiple other sarcoma types develop in children and adolescents and are even rarer than CCSK.^12^, ^13^, ^14^ As a result, these other primary renal sarcomas are treated either according to multimodal adult strategies or those adapted from instances when originating in other anatomic sites, such as Ewing sarcoma (EWS) that typically manifests in bone and soft tissues.^15^, ^16^, ^17^ Local disease control has been a principal objective for pediatric surgeons and oncologists when treating EWS but can be more challenging when the disease origin is visceral.^15^ Standardizing therapy regimens for the heterogeneity of primary renal sarcomas arising among all ages therefore is constrained, given histologic rarity, diversity, and often absent biologic characterization.^17^ Nevertheless, molecular classification of these unusual and protean kidney sarcomas is progressing rapidly, which will facilitate more accurate diagnosis, prognosis, and identification of novel candidate targets for precision therapy.^11^^,^^18^^,^^19^

The principal purposes of this study, therefore, were first to describe the spectrum of primary renal sarcomas arising in children and adolescents given newer histologic and molecular classification features, second to examine the unique patient and disease characteristics that impact survival, and third to provide a framework for more specific histologic and biologic classification of primary renal sarcomas of childhood and adolescence that soon will inform stricter therapeutic precision. The authors hypothesized that pediatric renal sarcomas associate with extensive treatment variability in the absence of distinct biologic risk-assignment to guide more precise therapies.

## Methods

### Study design and participants

32 member institutions of Pediatric Surgical Oncology Research Collaborative (PSORC) contributed data on patients 21 years and younger at diagnosis of and treatment for a primary renal sarcoma between January 1st, 2000, and November 30th, 2022. Sources for patient capture included institutional cancer registries, pathology databases, and disease queries through electronic medical records (EMR). ICD.9 codes 189.0 and 189.1 and ICD.10 codes C64 and C65 were used to capture any malignant neoplasm of kidney and renal pelvis. Search terms also included any mention of “sarcoma” in the patient EMR and pathology databases, as well as specific histologic types: CCSK, EWS, infantile fibrosarcoma, rhabdomyosarcoma, undifferentiated sarcoma, anaplastic sarcoma of the kidney, and synovial sarcoma. The EMR of all patients identified through the initial query then were reviewed methodically to determine the precise histologic diagnosis and assess study eligibility.

### Ethics

To generate robust patient data for this rare renal cancer category, the authors leveraged the PSORC. At time of study, this large North American research collaborative was comprised of 45-member pediatric children's hospitals across the United States and Canada. All studies executed through PSORC are thoroughly reviewed, critiqued, and approved at the investigator and leadership levels and then similarly through the Institutional Review Board (IRB) of the Cincinnati Children's Hospital Medical Center (CCHMC). Each participating member institution that contributes patient data then obtains institution-specific IRB approval. Data Use Agreements (DUA) are secured at each participating member institution to share data through a password protected REDCap database. For the purposes of this specific PSORC study, institutional IRB approval was obtained, as were institution-specific DUAs. Given the retrospective study design and de-identification of abstracted patient data, individual consent was not required.

### Data extraction

Patient data were abstracted from the EMR, including age at renal sarcoma diagnosis, demographics (i.e., sex, race, and ethnicity, as defined in the 2020 US Census Bureau), presenting symptoms, and any cancer predisposition syndrome. Disease characteristics included sarcoma histologic type, stage of disease at presentation, metastatic site if present, greatest tumor diameter on diagnostic imaging, tumor rupture, margin status, lymph node involvement, and somatic molecular aberrations. Treatment characteristics examined whether the patient was enrolled on a formal COG or other study, any surgical intervention (e.g., biopsy, partial or radical nephroureterectomy, tumor thrombectomy), chemotherapy regimen, neoadjuvant and/or adjuvant therapy, and exposure to radiation therapy. Event-free survival (EFS) was defined as time from diagnosis to first event, whether relapse or death, and overall survival (OS) was determined from date of diagnosis through last vital contact.

### Statistics

Frequency of each histologic sarcoma diagnosis was recorded, and descriptive statistics were performed to compare medians and interquartile ranges for continuous variables between histologies. Patient, disease, and treatment characteristics as categorical variables then were compared across sarcoma type using Pearson Chi-square and Kruskal–Wallis tests. For example, age at presentation of each sarcoma type was compared using Kruskal–Wallis test and displayed as box plots. Wilcoxon rank sum tests were used for comparisons of continuous variables between sarcoma types. Kaplan–Meier analyses were performed to plot 4-year and actuarial EFS and OS for specific patient, disease, and treatment characteristics. Cox regression analysis was performed to determine whether age impacted survival independently from histology. Initial descriptive data and Kaplan–Meier plots were performed by a biostatistician (HC) supported through the Vanderbilt Ingram Cancer Center and the Surgical Outcomes Center for Kids (SOCKs) at the Monroe Carell Jr. Children's Hospital at Vanderbilt using R Core Team statistical analysis software version 3.5.1. Additional analyses were conducted using SPSS Version 25.0.0. Statistical significance was set *a priori* at p < 0.05.

### Role of the funding source

There was no funding source for this study.

## Results

**Fig. 1:**
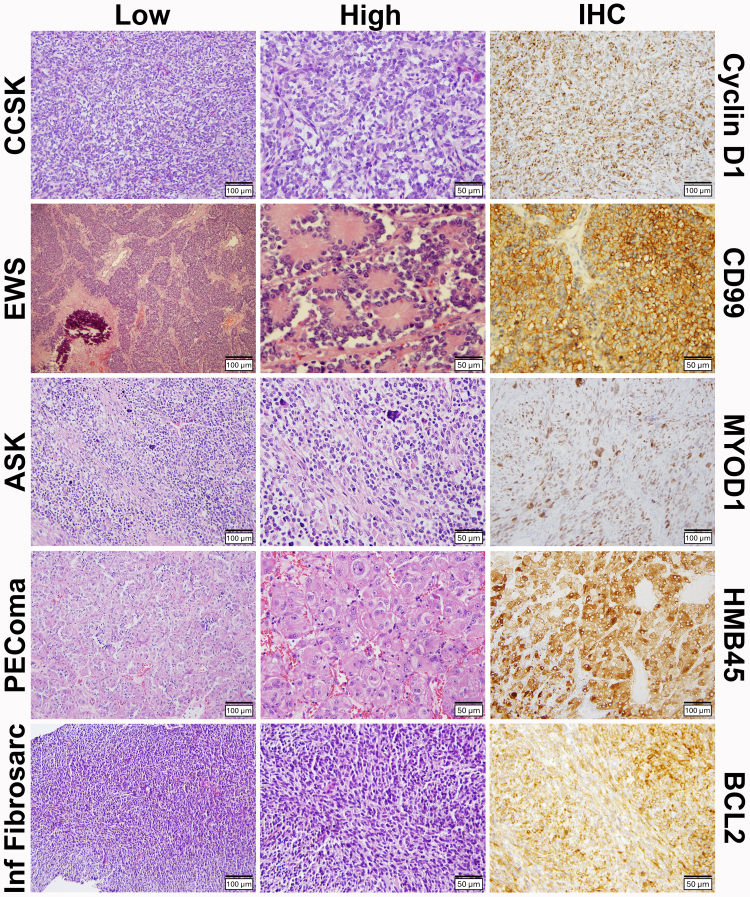
Representative histologic diversity of five pediatric renal sarcomas depicted in each row. Low (20×; 100 μm bar) and high (40×; 50 μm bar) power photomicrographs are shown in the first two columns, respectively, with an immunostain characteristic of the histology displayed in the third column (μm bars shown). *(Top row)* CCSK: clear cell sarcoma of the kidney and cyclin D1 detection. *(Second row)* EWS: Ewing sarcoma and CD99 detection. *(Third row)* ASK: anaplastic sarcoma of the kidney and MYOD1 (myogenic differentiation-1) detection.^14^ Note the large anaplastic nuclei in these photomicrographs. *(Fourth row)* PEComa: perivascular epithelioid cell neoplasm and HMB45 (Human Melanoma Black 45 monoclonal antibody) detection. *(Bottom row)* Inf Fibrosarc: infantile fibrosarcoma and BCL2 (B-cell lymphoma-2) detection.

**Fig. 2:**
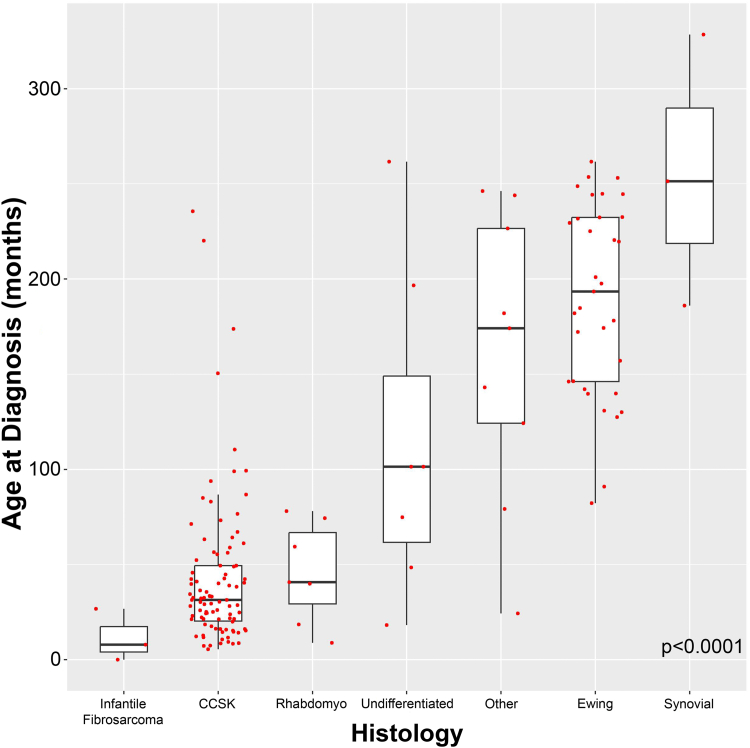
This figure shows the strong association of histology with age (months) at diagnosis.

Contributing PSORC institutions identified 158 patients diagnosed with and treated for a primary renal sarcoma before 22 years of age during the study period. Eleven unique sarcoma histologies were revealed (Fig. 1). As expected, CCSK (n = 94), EWS (n = 33), undifferentiated sarcoma (n = 8), and rhabdomyosarcoma (n = 7) predominated, while infantile fibrosarcoma and synovial sarcoma accounted for three cases each. Even rarer sarcoma types occurred infrequently and were pooled together as “other”, including “spindle cell neoplasms” or “renal sarcoma, not otherwise specified” (n = 4), DICER1-associated anaplastic sarcoma (n = 2), desmoplastic small round cell tumor (DSRCT; n = 2), malignant perivascular epithelioid cell tumor (PEComa, n = 1), and epithelioid sarcoma (n = 1). Among evaluated demographic features across sarcoma types, only age at diagnosis varied significantly with histology (p < 0.0001; Supplemental Table S1; Fig. 2). Neither sex, race, nor ethnicity associated significantly with histology (Supplemental Table S1). 11/155 (7.1%) carried a known cancer predisposition syndrome (Supplemental Table S1). DICER1 syndrome was present in three patients (3/158, 1.9%), two having an anaplastic sarcoma of the kidney and one an undifferentiated sarcoma (p = 0.0003). Li-Fraumeni syndrome was present in two patients, one each having infantile fibrosarcoma and rhabdomyosarcoma (p < 0.0001). Tuberous sclerosis complex was present in one patient having PEComa (p = 0.021). The remaining five patients having a cancer predisposition comprised the other germline pathogenic variants not previously associated with a renal sarcoma.

Presenting symptoms varied significantly with sarcoma type, particularly regarding abdominal pain (n = 55; p < 0.0001), palpable mass (n = 54; p = 0.013), and hematuria (n = 40; p < 0.0001; Supplemental Table S2). Only three patients presented with hypertension, which did not associate with sarcoma type (p = 0.91). Despite significant correlation of age at presentation with sarcoma type (p < 0.0001), greatest tumor dimension at diagnosis did not vary significantly with specific histology (p = 0.31; Supplemental Table S2). Laterality was evenly distributed across sarcoma types, and only three (3/153, 2%) patients presented with bilateral disease (p = 0.18; Supplemental Table S2). Disease staging (i.e., I-IV) at diagnosis was similar between sarcoma groups (p = 0.31). 45/151 (30%) patients presented with metastatic disease, which was statistically similar across sarcoma types (p = 0.28; Supplemental Table S2). However, pulmonary metastasis (n = 19/158; 12%), the most frequent site of hematogenous dissemination in this cohort, did vary significantly across sarcoma histology, occurring most commonly with EWS (p = 0.024; Supplemental Table S2). Bone metastasis was present at diagnosis in 12/158 (7.6%) patients, which occurred exclusively in and with similar frequencies between CCSK (n = 9/94; 9.6%) and EWS (n = 3/33; 9.1%; p = 0.79; Supplemental Table 2). Peritoneal sarcomatosis occurred exclusively in 3/94 (3.2%) patients with CCSK (p = 0.91). Only two patients (2/158, 1.3%), one each with CCSK and rhabdomyosarcoma, presented with brain metastases (p = 0.11). 21 patients (21/158, 13.3%) had regional lymph node involvement documented on pathology after upfront resection, and CCSK (n = 15/94, 16%) was the most common type, although differences across the other histologies were statistically similar (p = 0.83). Finally, of 81 (52%) renal sarcoma specimens tested, somatic or tumor level molecular alterations (n = 44; 54.3%) were detected only in EWS (n = 26/26; 100%), CCSK (n = 17/38; 44.7%), and rhabdomyosarcoma (n = 1/2; 50%; p = 0.001; Supplemental Table S3). For EWS, the most common recurring mutation was the *EWS-FLI1* fusion [t (11;22) (q24; q12)] (Ewing sarcoma breakpoint region 1–Friend leukemia virus integration 1; n = 23/26; 88.5%), whereas for CCSK, internal tandem duplications in the last exon of *BCOR* (n = 9/17; 52.9%) and translocation between *BCOR-CCNB3* (BCL6 co-repressor–cyclin B3 fusion; n = 3/17; 17.6%) predominated (Supplemental Table S3).

**Fig. 3:**
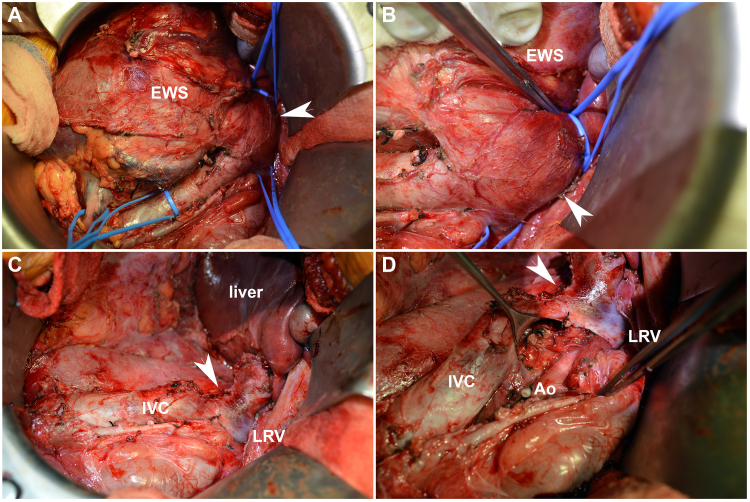
*(A, B)* This figure shows a right renal Ewing sarcoma (EWS) presenting in a 14-year-old with tumor thrombus extending to infrahepatic inferior vena cava (IVC). Blue tapes are for control of IVC and left renal vein (LRV). Arrowheads point to the tumor thrombus. *(C, D)* Arrowheads depict partial resection of IVC with thrombectomy and suture-line repair. Note empty tumor bed and post-dissection of pericaval and periaortic lymph nodes. (Ao, aorta; ligature on right renal artery).

Therapeutic strategies varied significantly across sarcoma types (p = 0.0008; Supplemental Table S4). Definitive surgical resection was accomplished in 131/150 (87.3%) patients, whereas 17/150 (11.3%) patients were treated medically only, which did not vary between sarcoma diagnoses (p = 0.45; Supplemental Table S4). As is typical for COG renal tumor protocols, if a newly discovered kidney mass in a child is deemed resectable on diagnostic imaging, upfront nephroureterectomy without preoperative biopsy is recommended and typically performed (Fig. 3). Considering the order of multimodal therapy among this cohort, 127/156 (81.4%) patients received initial nephroureterectomy (Supplemental Table S4; Fig. 3A–D). 16/156 (10.3%) patients received neoadjuvant chemotherapy with delayed tumor resection. 11/156 (7.1%) patients received only chemotherapy with the addition of radiation for local control. Specific treatment regimen and protocol for each patient when documented are shown in Supplemental Table S5. At the time of definitive resection, tumor thrombectomy (n = 9/158; 5.7%) was performed at significantly different frequencies according to histology, with EWS predominating (n = 6; p = 0.004; Fig. 3A–D). Renal vein (n = 6; p = 0.037) and inferior vena caval thrombi (n = 7; p = 0.001) were the most prevalent levels for tumor extension and again most frequent for EWS. Margin-negative (i.e., R0) resection was accomplished in 109/148 (73.6%) patients, which appeared to vary with sarcoma type (p = 0.05; Supplemental Table S4). Radiotherapy was included in the management of 111/151 (74%) patients, which also varied significantly across sarcoma types (p = 0.00018; Supplemental Table S4).

The presence of chronic health conditions affected 79/158 survivors (50%) and differed significantly across primary renal sarcoma type (p = 0.006; Supplemental Table S6). Chronic kidney disease (n = 26/158; 16%) was the most significant health issue affecting survivors and was most prevalent among EWS patients (n = 9/33; 27%; p = 0.041; Supplemental Table S6). Among the entire cohort, two patients were recorded as receiving dialysis and three a kidney transplant, all of whom were survivors of EWS. Second malignant neoplasms occurred in 7/140 survivors (5%) of their primary renal sarcoma, which varied significantly across histologic type (p = 0.024; Supplemental Table S6).

For the entire cohort, 115/145 (79.3%) patients were alive without evidence of disease at last contact, which differed significantly for the sarcoma types (p = 0.026). The median follow up for the cohort was 65 months (IQR: 29–122), which was not significantly different across sarcoma groups (p = 0.81; Supplemental Table S6). CCSK showed the highest absolute survival of the various histologies (n = 76/87; 87.4%). Among decedents (n = 13), cause of death was most associated with the primary renal sarcoma and disease progression, which occurred similarly across histologies (p = 0.35). While no perioperative deaths were documented, two CCSK patients died during cancer-directed therapy (Supplemental Table S6).

**Fig. 4:**
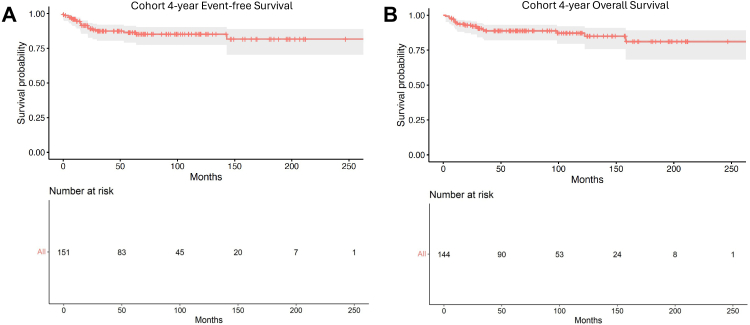
This figure shows 4-year EFS (event-free survival) *(A)* and OS (overall survival) *(B)* survival for the entire cohort.

**Fig. 5:**
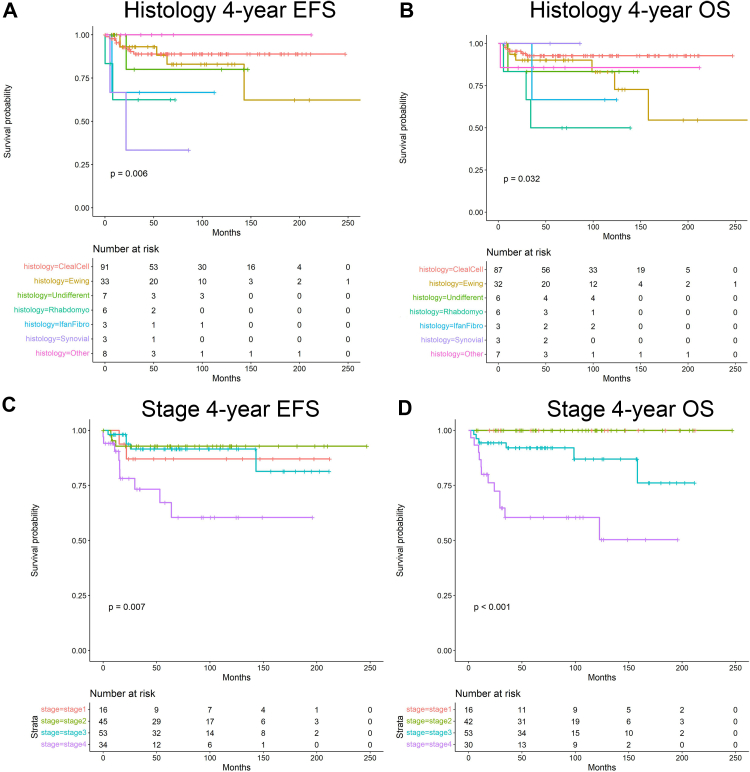
This figure shows 4-year EFS and OS for histology (i.e., sarcoma type; *A, B*) and then for stage of disease *(C, D)*.

**Fig. 6:**
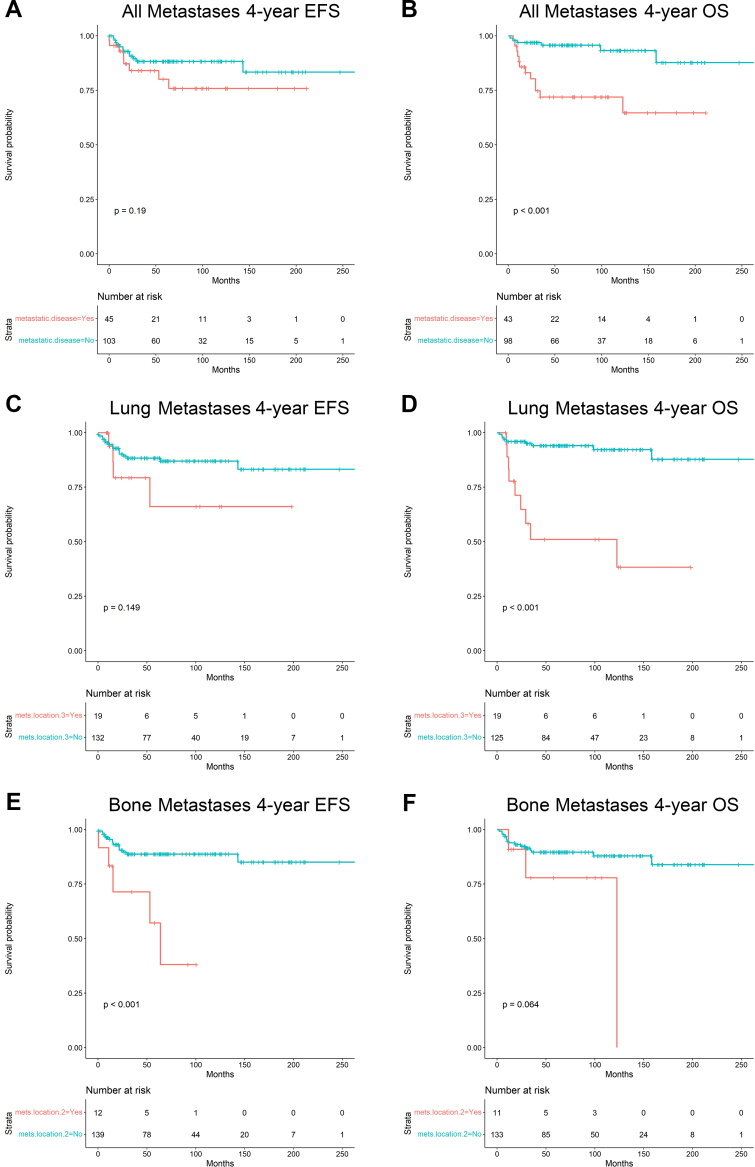
This figure shows impact of all metastases pooled *(A, B)*, lung metastases only *(C, D)*, and bone metastases only *(E, F)* on 4-year EFS and OS.

Using Kaplan–Meier analysis, 4-year EFS and OS were estimated for the entire cohort and then stratified by variables of interest. After pooling all renal sarcoma patients, 4-year EFS was 0.87 (95% CI 0.81–0.92), and OS was 0.89 (95% CI 0.82–0.93; Fig. 4). When comparing all sarcoma types, 4-year EFS and OS varied significantly with histology (p = 0.006 and p = 0.032, respectively; Fig. 5A, B). Corresponding survival estimates with 95% CIs are provided in Supplemental Table S7.1. When adjusting for specific histology and using CCSK as the reference, Cox regression showed a significantly increased Hazards ratio (HR) for rhabdomyosarcoma regarding both EFS (HR 5.25, 95% CI 1.12–24.53; p = 0.035) and OS (HR 9.36, 95% CI 2.30–38.11; p = 0.002; Supplemental Table S8). Stage at presentation was associated with significantly worse 4-year EFS (p = 0.0069) and OS (p < 0.0001) with increasing disease burden (Fig. 5C, D). Corresponding survival estimates with 95% CIs are provided in Supplemental Table S7.2. After pooling all lymphatic and hematogenous dissemination, presentation with any metastatic disease showed no significant difference for 4-year EFS (p = 0.19) but did reveal a significantly worse 4-year OS (p = 0.00017; Fig. 6A, B). Corresponding survival estimates with 95% CIs are provided in Supplemental Table S7.3. On separate analysis, lymphatic spread did not independently impact 4-year EFS or OS (p = 0.79 and p = 0.08, respectively; data not shown). However, metastatic site significantly impacted EFS and OS uniquely. Beginning with the most common hematogenous site, pulmonary metastases did not significantly affect 4-year EFS (0.79, 95% CI 0.49–0.93 vs 0.88, 95% CI 0.81–0.93; p = 0.149) but were associated with significantly worse OS (0.51, 95% CI 0.25–0.72 vs 0.94, 95% CI 0.88–0.97; p < 0.0001; Fig. 6C, D). Corresponding survival estimates with 95% CIs are provided in Supplemental Table S7.4. Among patients with bone metastases, 4-year EFS was significantly lower than in those without this dissemination (0.71, 95% CI 0.34–0.90 vs 0.89, 95% CI 0.82–0.93; p = 0.0004), whereas OS showed a similar numerical pattern—0.78 (95% CI 0.35–0.94) vs 0.90 (95% CI 0.83–0.94)— this difference was not statistically significant (p = 0.064; Fig. 6E, F). Corresponding survival estimates with 95% CIs are provided in Supplemental Table S7.5. Both patients having brain metastases died before 4 years, which impacted OS survival significantly (p < 0.0001).

The authors questioned whether somatic molecular alterations present in, and diagnostic of various renal sarcomas impacted 4-year survival. When pooled and compared against none detected, the presence of any tumor-level molecular alteration did not affect EFS or OS (p = 0.73 and p = 0.43, respectively). Corresponding survival estimates with 95% CIs are provided in Supplemental Table S7.6. When analyzed within a given histology, the presence of specific diagnostic molecular alterations also did not impact 4-year EFS or OS (CCSK: p = 0.91 and p = 0.12; EWS: p = 0.76 and p = 0.52; data not shown).

Regarding treatment strategy, 4-year EFS differed by order of therapy and was lowest among patients receiving neoadjuvant therapy followed by delayed nephrectomy (0.711 [95% CI 0.394–0.833]) compared with those undergoing upfront resection followed by adjuvant therapy (0.899 [95% CI 0.824–0.943]; p = 0.027), although 4-year OS did not differ significantly across groups (p = 0.69; Supplemental Table S7.7). Use of radiotherapy for local control did not significantly affect 4-year EFS (radiotherapy: 0.859 [95% CI 0.774–0.914] vs no radiotherapy: 0.915 [95% CI 0.758–0.972]; p = 0.66) or 4-year OS (radiotherapy: 0.909 [95% CI 0.831–0.951] vs no radiotherapy: 0.824 [95% CI 0.648–0.951]; p = 0.29; Supplemental Table S7.8). Neither race nor ethnic group showed differences in incidence or survival across the various sarcomas.

## Discussion

This study underscores the complex clinical and histologic heterogeneity of primary renal sarcomas presenting in childhood and adolescence, which, given their individual rarity, further complicates standardizing therapeutic strategies. In this large cohort of pediatric sarcomas collated from a North American research collaborative (PSORC), survival at 4 years appeared most dependent on the unique histology, stage of disease at presentation, and hematogenous metastatic pattern. Foremost, while sarcoma resection, whether radical nephroureterectomy or nephron-sparing partial nephrectomy, remains the cornerstone of treatment, multimodal regimens incorporating intensive chemotherapy and radiotherapy often are required to achieve local control and optimize overall survival. Notably, survival for children and adolescents having primary renal sarcomas as a disease category remains favorable, albeit less than that reported for the far more common Wilms tumor.^2^^,^^5^^,^^20^

The diversity of renal sarcoma histology that manifests in childhood and adolescence raises fascinating questions regarding the unique mesenchymal cells of origin, nephric developmental biology, and the timing of malignant transformation. Two of 11 histologies (i.e., CCSK and EWS) accounted for 127/158 (80.4%) pediatric sarcoma cases, whereas 9 distinctive histologies comprised the remaining 31/158 (19.6%) diagnoses over this two-decade study. Of demographic features evaluated, histology associated only with age at presentation. Notably, after controlling for histology, age was not associated with survival, inferring that sarcoma type is the principal outcome determinant. Indeed, this analysis showed significant association of histology with both EFS and OS at 4 years. With a median follow up of 65 months, CCSK and undifferentiated sarcomas showed the best actuarial survival (87.4% and 85.7%, respectively), whereas EWS (68.8%) and the other 8 histologies (ranging between 33.3% and 71.4%) showed the lowest long-term survival. The COG recently reported excellent outcomes for CCSK patients enrolled on umbrella protocol, AREN03B2, and treated on or according to the high-risk therapeutic trial, AREN0321.^5^ In that study, EFS and OS from CCSK at 4 years were 85.3% and 94.6%, respectively, which our findings mirrored (88.7% and 92.6%, respectively) and certainly included overlapping patients (see Acknowledgments). Taken together, these excellent results for CCSK in the current treatment era speak to the efficacy of multi-institutional cooperative trials to standardize therapy and optimize outcomes for rare pediatric cancers according to strict risk assignment.

Regarding primary renal EWS arising in children and adolescents, a paucity of case reports or series can be found in the published literature, and none emerges from a cooperative pediatric cancer group to guide standardized management.^13^^,^^16^^,^^21^ This PSORC cohort, therefore, represents the largest analyzed and reported to date for renal EWS arising in children and adolescents. Visceral presentations of extra-osseous EWS are exceedingly rare, although the kidney is a relatively common primary site, and metastases overall are more frequent with vital organ primaries when compared to musculoskeletal origins.^15^ Indeed, in our cohort, 37% of renal EWS presented with distant metastases, the most of any histology, and the most prevalent diagnosis to involve the lungs. Potentially complicating surgery, EWS was the most common histology in this cohort to present with tumor thrombus requiring a technically challenging inferior vena cava thrombectomy to accomplish optimal local control, which also has been documented in several case reports.^16^^,^^21^ Tumor thrombus is known to affect about 5–10% of renal tumors in children and adolescents, and cases need to be individualized regarding thrombectomy with initial or delayed nephrectomy for sarcoma histologies, as has been reported for Wilms tumor with venous thrombus.^22^ Notably, and novel from this PSORC cohort, renal presentation of EWS appears to associate with significant late health effects in long term survivors, which interestingly has been reported also for its osseous forms, and principally involved more severe chronic renal insufficiency, need for dialysis and kidney transplant, as well as second malignant neoplasms.^23^

As expected, increasing burden of disease at presentation bore out as a significant variable that challenged therapy and negatively impacted survival within this cohort. Specifically, primary pediatric renal sarcomas manifested multiple sites and patterns of hematogenous metastases that varied across histologic type. Specifically, CCSK presented with, in descending frequency, bone, pulmonary, peritoneal, hepatic, and intracranial metastases. This propensity for CCSK to metastasize widely has been documented previously.^5^^,^^9^^,^^24^^,^^25^ In contrast, EWS presented with pulmonary metastases most commonly, then bone. Three patients with rhabdomyosarcoma presented with either brain, pulmonary, or hepatic metastases, while only one patient with undifferentiated sarcoma presented with pulmonary metastases. Hence, metastatic patterns appeared heterogeneous across sarcoma type but with overlapping features too. While often clinically silent, imaging for extracranial metastases at diagnosis and then of common relapse sites are routinely and systematically performed before, during, and after therapy. The authors believe that dedicated brain imaging would follow symptom manifestation and that the numbers of this rare metastatic and relapse site revealed herein therefore are accurate.

The authors acknowledge several limitations with this study that temper rigorous conclusions. Foremost, the ideal study design to characterize rare pediatric cancers remains elusive. Beyond the recent report from the COG evaluating treatment of CCSK on the high-risk therapeutic trial, AREN0321, or within the context of its umbrella study, AREN03B2, no cooperative, multi-institutional, prospective trial has been published evaluating the remaining ∼40% of even scarcer pediatric renal sarcomas. Such a trial would have to be international in scope. National cancer databases, while appropriately powered, often lack critical detail (e.g., clinical, molecular, and histologic data) to differentiate rare pediatric diagnoses from others or to delineate specific therapeutic nuances to draw firm conclusions that impact or change current practices. Moreover, national cancer databases often represent only a sampling of patients having specific malignancies. As a result, the authors collectively believed that leveraging a large North American cancer research collaborative (i.e., PSORC) consisting of 45-member pediatric institutions would afford a blend of both power and detail not available through other investigative mechanisms. When comparing our observations with other reports, the authors feel confident in the data abstraction from 32 different institutions given specific similarities (e.g., age at diagnosis of sarcoma types, survival from CCSK consistent with COG, and metastatic patterns, among others; see Acknowledgments), which was standardized through a detailed data dictionary and included robust instructions and definitions. That acknowledged, nuances with unique clinical features, such as, what constitutes a relapse event vs a metastasis that persisted through therapy when reviewing a medical record retrospectively, could introduce ambiguity to interpretation and therefore affect specifically EFS, for example. The PSORC and primary study principal investigators (PI) were available to clarify investigator questions but did not have a specific mechanism for central review or verification of all data. The PSORC received all entered data, which were then cleaned and distributed to study PIs for analysis. Encouragingly, this retrospective collaborative study did not identify any race or ethnic group disparity in incidence or outcome regarding renal sarcomas. However, the study was not designed to investigate detailed social determinants of health that could impact disparate early or late health effects, as reported elsewhere for non-Wilms tumor malignancies.^26^ Finally, the rarity of these pediatric renal sarcomas likely introduced uncertainty with some histologic diagnoses, leading to vague terminology in pathology reports that could impact tumor classification and therefore alter study findings. Further, knowledge regarding the molecular underpinnings for these rare malignancies has been improving rapidly across the study period, which could introduce inaccuracies between sarcoma types. As a result, nomenclature over the study period has changed too. For example, the cellular variant of congenital mesoblastic nephroma is now also categorized as infantile fibrosarcoma of the kidney, which may have resulted in falsely lower case numbers despite its rare incidence.^27^ Indeed, an era effect likely existed with older cases presumably having less frequent molecular testing to diagnose these rare sarcomas more precisely or to define underlying predisposition syndromes. Similarly, comparing clinical data between the ultra-rare forms of pediatric renal sarcomas reported herein must be interpreted cautiously but were important to show for completeness. Summarily, however, the authors collectively believe that this multi-institutional study design that accrued patients over two decades, albeit retrospective, was the ideal mechanism to generate both sufficient power and detail to paint the most accurate and current landscape for the challenge of these rare pediatric renal sarcomas.

In summary, this large North American research collaborative study details the current state and heterogeneity of renal sarcomas arising in children and adolescents. Sarcoma type varies significantly with age at presentation, and metastatic patterns, including tumor thrombus predilection, tend to associate with specific histologies. The factors most deleteriously impacting 4-year survival were histology, disease burden at presentation, and metastatic patterns. An “all hands-on deck” multidisciplinary approach employing intensive multimodal therapy, including surgery, chemotherapy, and radiotherapy, is mandated to accomplish long-term survival. As germline and somatic tumor molecular testing evolve and introduce novel predispositions and targetable mutations, the authors anticipate better screening, and more precise therapies will emerge on the horizon. This study, therefore, should serve as a plea for better biologic classification of this rare renal tumor category that allows precise risk-stratification and standardization of multimodal, target-specific cancer care.

## Contributors

Dr. Harold N. Lovvorn, III (HNL) principally designed the statistical analysis, wrote the manuscript, incorporated all edits and suggestions from each contributing author, and prepared all figures. Dr. Lovvorn was responsible for the original submission and all revisions.

Dr. Elisabeth Tracy contributed equally to HNL to develop study aims, to prepare the analysis, and to edit the manuscript. Dr. Tracy has verified the underlying study data having provided an independent preliminary analysis to determine its validity.

Dr. Marcus Malek was a critical investigator to design the study aims and analyses and to edit the manuscript. Dr. Malek contributed equally to Drs. Lovvorn and Tracy for development of the study aims and design and for completion of the manuscript.

Dr. Katlyn G. McKay prepared all data for statistical analysis and was instrumental in data abstraction. She also presented elements of the data at the International Pediatric Surgical Oncology meeting combined with the American Pediatric Surgical Association in May 2024. Dr. McKay has independently verified the underlying study data as well.

Dr. Catherine Beckhorn contributed equally to Dr. McKay. Dr. Beckhorn also has verified the underlying study data, having performed preliminary analyses with Dr. Tracy.

Dr. Heidi Chen conducted the entire statistical analysis and has verified those data.

Dr. Roshni Dasgupta oversaw the entire analysis and directs the Pediatric Surgical Oncology Research Collaborative.

Dr. Timothy B. Lautz provided similar contributions to Dr. Dasgupta and edited the manuscript in detail.

Dr. Jennifer Aldrink provided significant leadership in conduct of the research and provided detailed edits to the manuscript.

Chloe Boehmer contributed significant administrative support for completion of the study and submission of the article.

All other authors contributed equally at their respective institutions to garner IRB approval, DUA completion, data abstraction, and data sharing. Also, all authors reviewed and provided individual edits and approval of the article through its various drafts.

## Data sharing statement

Deidentified individual participant data (IPD) underlying the results reported in this article, together with the study protocol, statistical analysis plan, and analytic code, will be made available by the Pediatric Surgical Oncology Research Collaborative (PSORC) to qualified investigators upon reasonable request, beginning at publication and for up to five years thereafter. Access is limited to methodologically sound proposals for research consistent with ethics approval and applicable laws. Data will be shared following review and approval by the PSORC leadership and execution of a legally approved data use agreement; additional institutional or ethics approvals may be required. Requests should be directed to the corresponding author on behalf of PSORC. Data that could compromise participant privacy or violate third-party rights will not be shared.

## Declaration of generative AI and AI-assisted technologies in the manuscript preparation process

During the final preparation of this work, the author, Harold N. Lovvorn, III, used ChatGPT PLUS to revise only the manuscript format on third submission to satisfy *Lancet Discovery Science eClinicalMedicine* requirements and specifically regarding how best to provide survival estimates according to *Lancet* standards. After using this tool/service, the author above reviewed and edited the suggested content as needed and takes full responsibility for the content of the published article.

## Declaration of interests

The authors have no conflicts of interest to report.
