# Supplementary material for: Renal sarcomas in children and adolescents: a retrospective, multicenter cohort study
Source: eClinicalMedicine. 2025 Dec 18;91:103713. doi: 10.1016/j.eclinm.2025.103713 (PMC12775861; doi:10.1016/j.eclinm.2025.103713)
Supplement: Supplementary Material [file mmc1.docx]

**Study group members as authors “on behalf of the Pediatric Surgical Oncology Research**

**Collaborative”:**

| **First names** | **Surnames** | **Degree(s)** | **Affiliation** |
| --- | --- | --- | --- |
| Heidi | Chen | Ph.D. | 1 |
| Harold J. | Leraas | M.D., M.S.P.H.S., M.H.S. | 2 |
| David W. | Hoyt | M.D. | 3 |
| Emily K. | Myers | M.D. | 4 |
| Nicolas G. | Cost | M.D. | 5 |
| Charles R. | Marchese | D.O. | 6 |
| Amanda R. | Jensen | M.D. | 6 |
| Michela M. | Carter | M.D. | 7 |
| John | Lundstedt | B.S. | 8 |
| Joseph G. | Brungardt | M.D. | 8 |
| Andrew M. | Davidoff | M.D. | 9 |
| Andrew J. | Murphy | M.D. | 9 |
| Sara | Mansfield | M.D. | 10 |
| Dave R. | Lal | M.D., M.P.H. | 11 |
| Jennifer M. | Schuh | M.D. | 12 |
| Sindhu V. | Mannava | M.D. | 13 |
| Adriana | Lopez | B.S. | 14 |
| Kelsey | Mello | M.A. | 14 |
| Shay | Rajaval | B.S. | 15 |
| Grace R. | Thompson | M.D. | 16 |
| Kathryn L. | Fowler | M.D. | 17 |
| Nathan | Martchenke | B.S. | 17 |
| Richard D. | Glick | M.D. | 18 |
| Kathleen | Doyle | M.D. | 19 |
| Paige | Abril | B.S. | 19 |
| Hannah N. | Rinehardt | M.D. | 20 |
| Jacob | Davidson | M.Sc. | 21 |
| Claire A. | Wilson | Ph.D. | 21 |
| Devashish | Joshi | M.D. | 22 |
| Michael | Stellon | M.D. | 22 |
| Alexandra | Dimmer | M.D. | 23 |
| Keyonna | Williams | M.D. | 24 |
| Maya | Hammoud | M.D., M.B.A. | 24 |
| Merit | Gorgy | B.S. | 25 |
| Julia G. | Debertin | M.D. | 26 |
| Alyssa | Stetson | M.D. | 27 |
| William G. | Lee | M.D. | 28 |
| Aaron | Barkhordar | B.S. | 28 |
| Lauren K. | Mayon | P.A. | 29 |
| Anastasia | Kahan | M.D. | 30 |
| Michael | Pitonak | B.S. | 31 |

**Affiliations:**

^1^ Department of Biostatistics, Vanderbilt University Medical Center, Nashville, TN

^2^ Department of Surgery, Duke University Medical Center, Durham, NC

^3^ Department of Surgery, Division of Pediatric Surgery, University of Utah, Primary Children's Hospital, Salt Lake City, UT

^4^ Department of Pediatric Surgery, Children’s Hospital of Colorado, Denver, CO

^5^ Department of Surgery, Division of Urology, University of Colorado School of Medicine, Surgical Oncology Program, Children’s Hospital of Colorado, Denver, CO

^6^ Children’s Mercy Hospital, Kansas City, MO

^7^ Department of Surgery, Division of Pediatric Surgery, Ann and Robert H. Lurie Children’s Hospital of Chicago, Northwestern University Feinberg School of Medicine, Chicago, IL

^8^ Division of Pediatric Surgery, Cincinnati Children's Hospital Medical Center, Cincinnati, OH

^9^ Department of Surgery, St. Jude Children's Research Hospital, Memphis, TN

^10^ Department of Surgery, Division of Pediatric Surgery, Nationwide Children’s Hospital, The Ohio State University College of Medicine, Columbus, OH

^11^ Division of Pediatric Surgery, Medical College of Wisconsin, Children's Wisconsin, Milwaukee, WI

^12^ Department of Surgery, Medical College of Wisconsin, Children’s Wisconsin, Milwaukee, WI

^13^ Division of Pediatric Surgery, Riley Hospital for Children, Indiana University School of Medicine, Indianapolis, IN

^14^ Valley Children's Hospital, Madera, CA

^15^ University of Florida College of Medicine, Gainesville, FL

^16^ Department of Surgery, University of Florida, Gainesville, FL

^17^ Department of Surgery, Oregon Health & Science University, Portland, OR

^18^ Division of Pediatric Surgery, Feinstein/Northwell, Cohen Children’s Medical Center, Hempstead, NY

^19^ Department of Surgery, University of California Davis, Sacramento, CA

^20^ Division of Pediatric General & Thoracic Surgery, UPMC Children's Hospital of Pittsburgh, Pittsburgh, PA

^21^ Division of Pediatric Surgery, Children’s Hospital, London Health Sciences Centre, London, ON, Canada

^22^ Department of Surgery, University of Wisconsin School of Medicine and Public Health, Madison, WI

^23^ Division of Pediatric Surgery, SUNY Upstate Medical University, Syracuse, NY

^24^ Division of Pediatric Surgery, C. S Mott Children's Hospital, University of Michigan, Ann Arbor, MI

^25^ New York Medical College, Valhalla, NY

^26^ Department of Surgery, Mayo Clinic, Rochester, MN

^27^ Massachusetts General Hospital, Boston, MA

^28^ Division of Pediatric Surgery, Cedars-Sinai Medical Center, Los Angeles, CA

^29^ Division of Pediatric Surgery, University of Texas, MD Anderson Cancer Center, Houston, TX

^30^ Department of Surgery, Icahn School of Medicine Mount Sinai, New York, NY

^31^ Department of Surgery, Children’s Health Children’s Medical Center, University of Texas Southwestern, Dallas, TX

**Supplemental Table 1. Cohort Description**

|  | **N** | **CCSK**  **(N=94)** | **Ewing**  **(N=33)** | **Undifferent**  **(N=8)** | **Rhabdomyo**  **(N=7)** | **Infan Fibro**  **(N=3)** | **Synovial**  **(N=3)** | **Other**  **(N=10)** | **Combined**  **(N=158)** | **Statistic** |
| --- | --- | --- | --- | --- | --- | --- | --- | --- | --- | --- |
| **Age (months)** | 156 | 20.3 **31.4** 49.4  (42.8 +/-4 0.4) | 146.1 **193.4** 232.4  (189.6 +/- 50.4) | 61.6 **101.3** 149.0  (114.6 +/- 85.6) | 29.2 **40.7** 66.8  (45.6 +/- 26.5) | 3.9 **7.8** 17.3  (11.5 +/- 13.8) | 218.7 **251.3** 289.8  (255.2 +/- 71.3) | 124.2 **174.1** 226.6  (160.4 +/- 75.9) | 24.5 **48.6** 143.8 (87.5+/- 81.8) | P<0.0001* |
| **Sex** | 157 |  |  |  |  |  |  |  |  | P=0.78 |
| Female |  | 50% (46) | 48% (16) | 50% (4) | 43% (3) | 0% (0) | 33% (1) | 50% (5) | 48% (75) |  |
| Male |  | 50% (47) | 52% (17) | 50% (4) | 57% (4) | 100% (3) | 67% (2) | 50% (5) | 52% (82) |  |
| **Race** | 158 |  |  |  |  |  |  |  |  | P=0.1 |
| Native Am. |  | 1.1% (1) | 3.0% (1) | 0.0% (0) | 14.3% (1) | 0.0% (0) | 0.0% (0) | 0.0% (0) | 1.9% (3) |  |
| Asian Am. |  | 3.2% (3) | 0.0% (0) | 0.0% (0) | 0.0% (0) | 0.0% (0) | 33.3% (1) | 0.0% (0) | 2.5% (4) |  |
| Black |  | 16.0% (15) | 3.0% (1) | 0.0% (0) | 0.0% (0) | 33.3% (1) | 0.0% (0) | 40.0% (4) | 13.3% (21) |  |
| Other |  | 3.2% (3) | 6.1% (2) | 0.0% (0) | 14.3% (1) | 0.0% (0) | 0.0% (0) | 0.0% (0) | 3.8% (6) |  |
| Unknown |  | 11.7% (11) | 3.0% (1) | 12.5% (1) | 14.3% (1) | 0.0% (0) | 0.0% (0) | 10.0% (1) | 9.5% (15) |  |
| White |  | 64.9% (61) | 84.8% (28) | 87.5% (7) | 57.1% (4) | 66.7% (2) | 66.7% (2) | 50.0% (5) | 69.0% (109) |  |
| **Ethnicity** | 158 |  |  |  |  |  |  |  |  | P=0.82 |
| Hispanic |  | 9.6% (9) | 12.1% (4) | 25.0% (2) | 0.0% (0) | 0.0% (0) | 0.0% (0) | 20.0% (2) | 10.8% (17) |  |
| Non-Hispanic |  | 75.5% (71) | 81.8% (27) | 62.5% (5) | 85.7% (6) | 100.0% (3) | 66.7% (2) | 70.0% (7) | 76.6% (121) |  |
| Unknown |  | 14.9% (14) | 6.1% (2) | 12.5% (1) | 14.3% (1) | 0.0% (0) | 33.3% (1) | 10.0% (1) | 12.7% (20) |  |
| **Predisposition** | 155 |  |  |  |  |  |  |  |  | P=0.096 |
| No |  | 72.8% (67) | 72.7% (24) | 57.1% (4) | 85.7% (6) | 66.7% (2) | 66.7% (2) | 70.0% (7) | 72.3% (112) |  |
| Yes |  | 4.3% (4) | 3.0% (1) | 14.3% (1) | 14.3% (1) | 33.3% (1) | 0.0% (0) | 30.0% (3) | 7.1% (11) |  |
| Unknown |  | 22.8% (21) | 24.2% (8) | 28.6% (2) | 0.0% (0) | 0.0% (0) | 33.3% (1) | 0.0% (0) | 20.6% (32) |  |
| **Syndrome** | 158 |  |  |  |  |  |  |  |  |  |
| DICER1 |  | 0.0% (0) | 0.0% (0) | 12.5% (1) | 0.0% (0) | 0.0% (0) | 0.0% (0) | 20.0% (2) | 1.9% (3) | P=0.0003 |
| Li-Fraumeni |  | 0.0% (0) | 0.0% (0) | 0.0% (0) | 14.3% (1) | 33.3% (1) | 0.0% (0) | 0.0% (0) | 1.3% (2) | P<0.0001 |
| Tuberous Sclerosis |  | 0.0% (0) | 0.0% (0) | 0.0% (0) | 0.0% (0) | 0.0% (0) | 0.0% (0) | 10.0% (1) | 0.6% (1) | P=0.021 |

CCSK: clear cell sarcoma of the kidney. Ewing: Ewing sarcoma. Undifferent: undifferentiated sarcoma. Rhabdomyo: rhabdomyosarcoma. Infan Fibro: infantile fibrosarcoma. Other sarcomas include: “spindle cell neoplasms” or “renal sarcoma”, not otherwise specified (n=4), DICER1-associated anaplastic sarcoma (n=2), desmoplastic small round cell tumor (n=2), malignant perivascular epithelioid cell tumor (PEComa, n=1), and epithelioid sarcoma (n=1). Descriptive Statistics (N = 158). a **b** c represent the lower quartile, a, the median, **b**, and the upper quartile, c, for continuous variables. N is the number of non-missing values. Numbers after proportions are frequencies. Tests used: ^*^Kruskal-Wallis and Pearson tests (all others).

**Supplemental Table 2. Disease Characteristics at Presentation**

|  | **N** | **CCSK**  **(N=94)** | **Ewing**  **(N=33)** | **Undifferent**  **(N=8)** | **Rhabdomyo**  **(N=7)** | **Infan Fibro**  **(N=3)** | **Synovial**  **(N=3)** | **Other**  **(N=10)** | **Combined**  **(N=158)** | **Statistic** |
| --- | --- | --- | --- | --- | --- | --- | --- | --- | --- | --- |
| **Greatest tumor**  **Dimension (cm)** | 143 | 10.0 **11.4** 14.0  (11.9 +/- 3.6) | 7.0 **9.7** 12.1  (10.0 +/- 4.2) | 8.7 **10.5** 13.0  (11.1+/- 2.7 | 9.8 **11.0** 13.0  (10.6 +/- 4.8) | 10.0 **11.5** 13.0  (11.5 +/- 4.1) | 9.0 **12.3** 14.6  (11.7 +/- 5.7) | 5.3 **7.9** 14.8  (12.0 +/- 8.8) | 8.8 **11.0** 13.8  (11.4 +/- 4.2) | *P=0.31 |
| **Laterality** | 153 |  |  |  |  |  |  |  |  | P=0.18 |
| Right |  | 54% (49) | 48% (16) | 38% (3) | 50% (3) | 33% (1) | 33% (1) | 67% (6) | 52% (79) |  |
| Left |  | 46% (42) | 48% (16) | 62% (5) | 33% (2) | 67% (2) | 67% (2) | 22% (2) | 46% (71) |  |
| Bilateral |  | 0% (0) | 3% (1) | 0% (0) | 17% (1) | 0% (0) | 0% (0) | 11% (1) | 2% (3) |  |
| **Symptoms** | 158 |  |  |  |  |  |  |  |  |  |
| Hematuria |  | 15% (14) | 64% (21) | 25% (2) | 0% (0) | 0% (0) | 33% (1) | 20% (2) | 25% (40) | P<0.0001 |
| Abdominal pain |  | 21% (20) | 67% (22) | 38% (3) | 57% (4) | 0% (0) | 100% (3) | 30% (3) | 35% (55) | P<0.0001 |
| Palpable mass |  | 44% (41) | 12% (4) | 50% (4) | 43% (3) | 33% (1) | 0% (0) | 10% (1) | 34% (54) | P=0.013 |
| Distension |  | 31.9% (30) | 9.1% (3) | 12.5% (1) | 28.6% (2) | 33.3% (1) | 33.3% (1) | 0.0% (0) | 24.1% (38) | P=0.078 |
| **Tumor Rupture** | 149 |  |  |  |  |  |  |  |  | P=0.24 |
| No |  | 84.6% (77) | 81.2% (26) | 71.4% (5) | 40.0% (2) | 66.7% (2) | 66.7% (2) | 87.5% (7) | 81.2% (121) |  |
| Yes |  | 11.0% (10) | 6.2% (2) | 14.3% (1) | 40.0% (2) | 33.3% (1) | 0.0% (0) | 0.0% (0) | 10.7% (16) |  |
| Indeterminate |  | 4.4% (4) | 12.5% (4) | 14.3% (1) | 20.0% (1) | 0.0% (0) | 33.3% (1) | 12.5% (1) | 8.1% (12) |  |
| **Stage** | 152 |  |  |  |  |  |  |  |  | P=0.11 |
| Stage 1 |  | 8.7% (8) | 9.4% (3) | 42.9% (3) | 0.0% (0) | 0.0% (0) | 0.0% (0) | 30.0% (3) | 11.2% (17) |  |
| Stage 2 |  | 32.6% (30) | 31.2% (10) | 14.3% (1) | 16.7% (1) | 66.7% (2) | 50.0% (1) | 10.0% (1) | 30.3% (46) |  |
| Stage 3 |  | 42.4% (39) | 25.0% (8) | 14.3% (1) | 33.3% (2) | 33.3% (1) | 50.0% (1) | 30.0% (3) | 36.2% (55) |  |
| Stage 4 |  | 16.3% (15) | 34.4% (11) | 28.6% (2) | 50.0% (3) | 0.0% (0) | 0.0% (0) | 30.0% (3) | 22.4% (34) |  |
| **Metastases** | 151 |  |  |  |  |  |  |  |  | P=0.28 |
| No |  | 70% (63) | 61% (20) | 88% (7) | 50% (3) | 100% (3) | 100% (3) | 88% (7) | 70% (106) |  |
| Yes |  | 30% (27) | 39% (13) | 12% (1) | 50% (3) | 0% (0) | 0% (0) | 12% (1) | 30% (45) |  |
| **Metastatic Site** | 158 |  |  |  |  |  |  |  |  |  |
| Brain |  | 1.1% (1) | 0.0% (0) | 0.0% (0) | 14.3% (1) | 0.0% (0) | 0.0% (0) | 0.0% (0) | 1.3% (2) | P=0.11 |
| Bone |  | 9.6% (9) | 9.1% (3) | 0.0% (0) | 0.0% (0) | 0.0% (0) | 0.0% (0) | 0.0% (0) | 7.6% (12) | P=0.79 |
| Lung |  | 7.4% (7) | 30.3% (10) | 12.5% (1) | 14.3% (1) | 0.0% (0) | 0.0% (0) | 0.0% (0) | 12.0% (19) | P=0.024 |
| Liver |  | 2.1% (2) | 0.0% (0) | 0.0% (0) | 14.3% (1) | 0.0% (0) | 0.0% (0) | 0.0% (0) | 1.9% (3) | P=0.33 |
| Peritoneum |  | 3.2% (3) | 0.0% (0) | 0.0% (0) | 0.0% (0) | 0.0% (0) | 0.0% (0) | 0.0% (0) | 1.9% (3) | P=0.91 |
| Lymph nodes  (regional) |  | 16% (15) | 12% (4) | 0% (0) | 14% (1) | 0% (0) | 0% (0) | 10% (1) | 13% (21) | P=0.83 |

CCSK: clear cell sarcoma of the kidney. Ewing: Ewing sarcoma. Undifferent: undifferentiated sarcoma. Rhabdomyo: rhabdomyosarcoma. Infan Fibro: infantile fibrosarcoma. Other sarcomas include: “spindle cell neoplasms” or “renal sarcoma”, not otherwise specified (n=4), DICER1-associated anaplastic sarcoma (n=2), desmoplastic small round cell tumor (n=2), malignant perivascular epithelioid cell tumor (PEComa, n=1), and epithelioid sarcoma (n=1). Descriptive Statistics (N = 158). a **b** c represent the lower quartile, a, the median, **b**, and the upper quartile, c, for continuous variables. N is the number of non-missing values. Numbers after proportions are frequencies. Tests used: ^*^Kruskal-Wallis and Pearson tests (all others).

**Supplemental Table 3. Pathology and Biology**

|  | **N** | **CCSK**  **(N=94)** | **Ewing**  **(N=33)** | **Undifferent**  **(N=8)** | **Rhabdomyo**  **(N=7)** | **Infan Fibro**  **(N=3)** | **Synovial**  **(N=3)** | **Other**  **(N=10)** | **Combined**  **(N=158)** | **Statistic** |
| --- | --- | --- | --- | --- | --- | --- | --- | --- | --- | --- |
| **Residual Tumor** | 149 |  |  |  |  |  |  |  |  | P=0.52 |
| None (R0) |  | 71.9% (64) | 78.8% (26) | 71.4% (5) | 33.3% (2) | 33.3% (1) | 66.7% (2) | 62.5% (5) | 70.5% (105) |  |
| Microscopic (R1) |  | 9.0% (8) | 12.1% (4) | 0.0% (0) | 16.7% (1) | 33.3% (1) | 33.3% (1) | 25.0% (2) | 11.4% (17) |  |
| Gross (R2) |  | 5.6% (5) | 0.0% (0) | 0.0% (0) | 16.7% (1) | 0.0% (0) | 0.0% (0) | 0.0% (0) | 4.0% (6) |  |
| Unknown |  | 13.5% (12) | 9.1% (3) | 28.6% (2) | 33.3% (2) | 33.3% (1) | 0.0% (0) | 12.5% (1) | 14.1% (21) |  |
| **Margins** | 148 |  |  |  |  |  |  |  |  | P=0.05 |
| Negative |  | 77.8% (70) | 72.7% (24) | 57.1% (4) | 40.0% (2) | 50.0% (1) | 66.7% (2) | 75.0% (6) | 73.6% (109) |  |
| Positive |  | 16.7% (15) | 18.2% (6) | 0.0% (0) | 40.0% (2) | 0.0% (0) | 33.3% (1) | 25.0% (2) | 17.6% (26) |  |
| Unknown |  | 5.6% (5) | 9.1% (3) | 42.9% (3) | 20.0% (1) | 50.0% (1) | 0.0% (0) | 0.0% (0) | 8.8% (13) |  |
| **Tumor Rupture** | 149 |  |  |  |  |  |  |  |  | P=0.24 |
| No |  | 84.6% (77) | 81.2% (26) | 71.4% (5) | 40.0% (2) | 66.7% (2) | 66.7% (2) | 87.5% (7) | 81.2% (121) |  |
| Yes |  | 11.0% (10) | 6.2% (2) | 14.3% (1) | 40.0% (2) | 33.3% (1) | 0.0% (0) | 0.0% (0) | 10.7% (16) |  |
| Indeterminate |  | 4.4% (4) | 12.5% (4) | 14.3% (1) | 20.0% (1) | 0.0% (0) | 33.3% (1) | 12.5% (1) | 8.1% (12) |  |
| **Molecular Analysis** | 156 |  |  |  |  |  |  |  |  | P=0.001 |
| No |  | 60% (56) | 21.2% (7) | 57% (4) | 71% (5) | 0% (0) | 33% (1) | 30% (3) | 48% (75) |  |
| Yes |  | 40% (38) | 78.8% (26) | 43% (3) | 29% (2) | 100% (3) | 67% (2) | 70% (7) | 52% (81) |  |
| **Molecular**  **Alterations** | 81 |  |  |  |  |  |  |  |  | P<0.0001 |
| No |  | 55.3% (21) | 0% (0) | 100% (3) | 50% (1) | 100% (3) | 100% (2) | 100% (7) | 45.7% (37) |  |
| Yes |  | 44.7% (17) | 100% (26) | 0% (0) | 50% (1) | 0% (0) | 0% (0) | 0% (0) | 54.3% (44) |  |
| Internal Tandem Duplications BCOR |  | 52.9% (9) |  |  |  |  |  |  |  |  |
| BCOR-CCNB3 translocation |  | 17.6% (3) |  |  |  |  |  |  |  |  |
| Other^ |  | 29.4% (5)* |  |  |  |  |  |  |  |  |
| EWSR-FLI1  [t(11;22) (q24;q12] |  |  | 88.5% (23) |  |  |  |  |  |  | P<0.0001 |
| Other^ |  |  | 11.5% (3)** |  | 50% (1)# |  |  |  |  | P=0.0001 |

CCSK: clear cell sarcoma of the kidney. Ewing: Ewing sarcoma. Undifferent: undifferentiated sarcoma. Rhabdomyo: rhabdomyosarcoma. Infan Fibro: infantile fibrosarcoma. Other sarcomas include: “spindle cell neoplasms” or “renal sarcoma”, not otherwise specified (n=4), DICER1-associated anaplastic sarcoma (n=2), desmoplastic small round cell tumor (n=2), malignant perivascular epithelioid cell tumor (PEComa, n=1), and epithelioid sarcoma (n=1). Descriptive Statistics (N = 158). N is the number of non-missing values. Numbers after proportions are frequencies. Tests used: Pearson Chi-square tests. Gray shade indicates not documented.

*: Other CCSK mutations identified included: (Xp22.33-Xq28) present, a 15.1 kb questionable mosaic duplication from 1p (1p36.13), gain of 1q and loss of 22q, translocation from the between the long arms of chromosome 21 and 22 on FISH, tert promoter mutation, and several germline variants with potential clinical significance.

**: Other EWS mutations identified included: EWSR1 exon 7, FLI1 exon 7, loss of one copy of EWS gene locus

#: Other Rhabdomyosarcoma mutation identified: gain of one extra copy each of chromosomes Y, 2, 6, 12, 14, 17 and 18.

**Supplemental Table 4. Multimodal Therapies**

|  | **N** | **CCSK**  **(N=94)** | **Ewing**  **(N=33)** | **Undifferent**  **(N=8)** | **Rhabdomyo**  **(N=7)** | **Infan Fibro**  **(N=3)** | **Synovial**  **(N=3)** | **Other**  **(N=10)** | **Combined**  **(N=158)** | **Statistic** |
| --- | --- | --- | --- | --- | --- | --- | --- | --- | --- | --- |
| **COG Protocol** | 140 |  |  |  |  |  |  |  |  | P=0.61 |
| No |  | 26% (22) | 33% (11) | 50% (4) | 50% (3) | 0% (0) | 50% (1) | 40% (2) | 31% (43) |  |
| Yes |  | 74% (63) | 67% (22) | 50% (4) | 50% (3) | 100% (1) | 50% (1) | 60% (3) | 69% (97) |  |
| **Surgery Order** | 156 |  |  |  |  |  |  |  |  | P=0.0008 |
| Non-surgical |  | 0.0% (0) | 0.0% (0) | 0.0% (0) | 14.3% (1) | 0.0% (0) | 0.0% (0) | 12.5% (1) | 1.3% (2) |  |
| Neoadjuvant & delayed surgery |  | 8.5% (8) | 9.1% (3) | 12.5% (1) | 42.9% (3) | 33.3% (1) | 0.0% (0) | 0.0% (0) | 10.3% (16) |  |
| Initial surgery |  | 87.2% (82) | 72.7% (24) | 87.5% (7) | 42.9% (3) | 66.7% (2) | 66.7% (2) | 87.5% (7) | 81.4% (127) |  |
| Neoadjuvant, XRT, & surgery |  | 4.3% (4) | 18.2% (6) | 0.0% (0) | 0.0% (0) | 0.0% (0) | 33.3% (1) | 0.0% (0) | 7.1% (11) |  |
| **Initial Surgery** |  |  |  |  |  |  |  |  |  |  |
| Biopsy | 158 | 11% (10) | 24% (8) | 50% (4) | 43% (3) | 0% (0) | 0% (0) | 20% (2) | 17% (27) | P=0.023 |
| Partial Neph | 158 | 1.1% (1) | 6.1% (2) | 0.0% (0) | 0.0% (0) | 0.0% (0) | 0.0% (0) | 0.0% (0) | 1.9% (3) | P=0.67 |
| Total Neph | 158 | 89% (84) | 73% (24) | 88% (7) | 29% (2) | 67% (2) | 100% (3) | 40% (4) | 80% (126) | P<0.0001 |
| Debulk -  gross total | 158 | 1.1% (1) | 0.0% (0) | 0.0% (0) | 14.3% (1) | 33.3% (1) | 0.0% (0) | 0.0% (0) | 1.9% (3) | P=0.0008 |
| **Definitive**  **Surgery** | 150 |  |  |  |  |  |  |  |  | P=0.45 |
| No |  | 12.9% (12) | 9.7% (3) | 12.5% (1) | 16.7% (1) | 0.0% (0) | 0.0% (0) | 0.0% (0) | 11.3% (17) |  |
| Yes |  | 87.1% (81) | 87.1% (27) | 75.0% (6) | 83.3% (5) | 100.0% (3) | 100.0% (3) | 100.0% (6) | 87.3% (131) |  |
| Unknown |  | 0.0% (0) | 3.2% (1) | 12.5% (1) | 0.0% (0) | 0.0% (0) | 0.0% (0) | 0.0% (0) | 1.3% (2) |  |
| **Tumor**  **Thrombectomy** | 158 |  |  |  |  |  |  |  |  | P=0.004 |
| No |  | 98.9% (93) | 81.8% (27) | 100.0% (8) | 100.0% (7) | 100.0% (3) | 66.7% (2) | 90.0% (9) | 94.3% (149) |  |
| Yes |  | 1.1% (1) | 18.2% (6) | 0.0% (0) | 0.0% (0) | 0.0% (0) | 33.3% (1) | 10.0% (1) | 5.7% (9) |  |
| **Lymph Nodes** | 158 |  |  |  |  |  |  |  |  | P=0.017 |
| No |  | 7.4% (7) | 27.3% (9) | 37.5% (3) | 14.3% (1) | 33.3% (1) | 0.0% (0) | 0.0% (0) | 13.3% (21) |  |
| Yes |  | 92.6% (87) | 72.7% (24) | 62.5% (5) | 85.7% (6) | 66.7% (2) | 100.0% (3) | 100.0% (10) | 86.7% (137) |  |
| **XRT** | 151 |  |  |  |  |  |  |  |  | P=0.00018 |
| No |  | 14% (13) | 56% (18) | 25% (2) | 33% (2) | 67% (2) | 0% (0) | 43% (3) | 26% (40) |  |
| Yes |  | 86% (79) | 44% (14) | 75% (6) | 67% (4) | 33% (1) | 100% (3) | 57% (4) | 74% (111) |  |
| **XRT Sites** | 109 |  |  |  |  |  |  |  |  | P<0.0001 |
| Flank |  | 70.1% (54) | 42.9% (6) | 50.0% (3) | 0.0% (0) | 0.0% (0) | 33.3% (1) | 75.0% (3) | 61.5% (67) |  |
| Whole Abdomen |  | 19.5% (15) | 14.3% (2) | 16.7% (1) | 25.0% (1) | 100.0% (1) | 0.0% (0) | 0.0% (0) | 18.3% (20) |  |
| Whole Lung |  | 0.0% (0) | 21.4% (3) | 0.0% (0) | 0.0% (0) | 0.0% (0) | 0.0% (0) | 0.0% (0) | 2.8% (3) |  |
| Metastases |  | 1.3% (1) | 7.1% (1) | 16.7% (1) | 0.0% (0) | 0.0% (0) | 0.0% (0) | 0.0% (0) | 2.8% (3) |  |
| Other |  | 5.2% (4) | 14.3% (2) | 16.7% (1) | 75.0% (3) | 0.0% (0) | 0.0% (0) | 25.0% (1) | 10.1% (11) |  |
| Unknown |  | 3.9% (3) | 0.0% (0) | 0.0% (0) | 0.0% (0) | 0.0% (0) | 66.7% (2) | 0.0% (0) | 4.6% (5) |  |

CCSK: clear cell sarcoma of the kidney. Ewing: Ewing sarcoma. Undifferent: undifferentiated sarcoma. Rhabdomyo: rhabdomyosarcoma. Infan Fibro: infantile fibrosarcoma. Other sarcomas include: “spindle cell neoplasms” or “renal sarcoma”, not otherwise specified (n=4), DICER1-associated anaplastic sarcoma (n=2), desmoplastic small round cell tumor (n=2), malignant perivascular epithelioid cell tumor (PEComa, n=1), and epithelioid sarcoma (n=1). Descriptive Statistics (N = 158). N is the number of non-missing values. Numbers after proportions are frequencies. Tests used: Pearson Chi-square tests. Neph: nephroureterectomy. XRT: radiotherapy.

**Supplemental Table 5. Therapeutic Protocol and/or Regimen**

| **Histology per patient** | **Protocol** | **Regimen if not on Protocol** |
| --- | --- | --- |
| CCSK |  | Per AREN0321: VCR, DOXO, CPM, CARBO+ETOP (CyCE/VDCy) |
| CCSK | AREN0321 |  |
| CCSK |  | Per AREN0321: VCR, DOXO, CPM, CARBO+ETOP (CyCE/VDCy) |
| CCSK |  | Per AREN0321: VCR, DOXO, CPM, CARBO+ETOP (CyCE/VDCy) |
| CCSK | AREN0321 |  |
| CCSK | AREN0321, regimen I |  |
| CCSK |  | Per AREN0321 regimen I: CPMI, DOXO, VCR, ETOP |
| CCSK | EE4A |  |
| CCSK | AREN0321, regimen I |  |
| CCSK | AREN0321, regimen I |  |
| CCSK | AREN0321, regimen I |  |
| CCSK | NWTS5, regimen I |  |
| CCSK | AREN0321, regimen I |  |
| CCSK | AREN0321 |  |
| CCSK | AREN0321, UH1 |  |
| CCSK | AREN0321, UH1 |  |
| CCSK | AREN0321, regimen I |  |
| CCSK | NWTS5, regimen I |  |
| CCSK | AREN0321 |  |
| CCSK | AREN0321 |  |
| CCSK | AREN0321 |  |
| CCSK | AREN0321 |  |
| CCSK | AREN0321 |  |
| CCSK |  | Vincristine, doxorubicin, cytoxan |
| CCSK | AREN0321 |  |
| CCSK | AREN0321, UH1 |  |
| CCSK | AREN0321 |  |
| CCSK |  | ADR, CTX, VP16, VCR; CCG 4941, per regimen I |
| CCSK |  | NWTS5 VCR, Cytoxan, VP16, Doxorubicin, Actinomycin |
| CCSK | AREN03B2 and AREN0321 |  |
| CCSK | AREN0321, regimen I |  |
| CCSK |  | Per AREN0321, regimen I:  Cytoxan, VCR, Doxorubicin, Etoposide. |
| CCSK |  | cyclophosphamide, doxorubicin, vincristine, etoposide, ifosfamide, dactinomycin |
| CCSK | AREN0321, Regimen I |  |
| CCSK | AREN0321 |  |
| CCSK | AREN0321 |  |
| CCSK |  | Per regimen I: Doxo, VCR, Cytoxan, etoposide |
| CCSK | AREN0321, Regimen I |  |
| CCSK | AREN0321 |  |
| CCSK | AREN0321 |  |
| CCSK | AREN0321, Regimen I |  |
| CCSK |  | Per AREN0321, regimen I |
| CCSK | AREN0321 |  |
| CCSK | AREN0321 |  |
| CCSK | NWTS5 |  |
| CCSK | POG 9440 |  |
| CCSK | AREN0321 -> POG9964 |  |
| CCSK | AREN0321 |  |
| CCSK | AREN0321 |  |
| CCSK |  | Doxorubicin, Etoposide, Cyclophosphamide |
| CCSK | AREN03B2 |  |
| CCSK |  | Doxorubicin, Etoposide, Cyclophosphamide |
| CCSK | AREN0321 |  |
| CCSK | AREN0321 |  |
| CCSK | AREN0321 |  |
| CCSK | AREN0321 |  |
| CCSK | AREN0321 |  |
| CCSK | AREN0321, Regimen I |  |
| CCSK |  | Per AREN0321 |
| CCSK |  | Doxo, VCR, Cytoxan, Carboplatin, and Etoposide. |
| CCSK |  | NWTS5, Regimen I  vincristine, doxorubicin, etoposide, cyclophosphamide |
| CCSK |  | Per AREN0321, regimen I |
| CCSK |  | Per EE-4A -> AREN0321, regimen I |
| CCSK |  | Per AREN0321, regimen 1 |
| CCSK |  | Per NWTS5, regimen I |
| CCSK | DD4A per CCG4941 |  |
| CCSK | AREN0321 |  |
| CCSK | AREN0321 |  |
| CCSK | AREN0321, UH1 |  |
| CCSK | AREN0321 |  |
| CCSK |  | Doxorubicin, cyclophosphamide, vincristine, etoposide |
| CCSK | NWTS5, Regimen I, CCG4941 |  |
| CCSK | AREN0321, Regimen I |  |
| CCSK | AREN0321 |  |
| CCSK | NWTS5, Regimen I, CCG4941 |  |
| CCSK | AREN0321 |  |
| CCSK | AREN03B2 |  |
| CCSK | AREN0321 |  |
| CCSK | AREN0534 |  |
| CCSK | NWTS5, CCG 4941, regimen I |  |
| CCSK | CCG 4941 regimen I |  |
| CCSK | AREN0321 |  |
| CCSK |  | vincristine, doxorubicin, cyclophosphamide, and etoposide |
| CCSK |  | Regimen I: VCR/DOX/Cyclophos/Etoposide |
| CCSK |  | Regimen I: vincristine, doxorubicin, cyclophosphamide |
| EWS | AREN03B2 |  |
| EWS | AEWS0031: VDC/IE |  |
| EWS |  | [Vincristine, Adriamycin, cyclophosphamide alternating with ifosfamide/etoposide](https://ascopubs.org/doi/full/10.1200/JCO.2011.41.5703:) |
| EWS | AEWS0031 |  |
| EWS | AEWS0031 |  |
| EWS | AEWS0031 |  |
| EWS |  | topotecan/cyclophosphamide |
| EWS | AEWS0031 |  |
| EWS | CCG Protocol 7492, regimen A |  |
| EWS | AEWS0031 |  |
| EWS | AEWS1031, Arm A |  |
| EWS |  | Per AEWS1031, POG 9579 |
| EWS | AEWS0031 |  |
| EWS |  | CAV/IE |
| EWS | AEWS0031 |  |
| EWS |  | VADRIAC |
| EWS |  | VDC |
| EWS | AEWS0031 |  |
| EWS |  | AEWS1221 roadmap: VDC/IE, regimen A |
| EWS | AEWS1031 |  |
| EWS |  | NPTP with VDC/IE |
| EWS |  | ESFT13, Group B, high risk arm |
| EWS | AEWS0031 |  |
| EWS | AEWS0031 |  |
| EWS | AEWS1031 |  |
| EWS |  | Actinomycin, Vincristine, doxorubicin, cyclophosphamide, ifosfamide and etoposide |
| EWS | AEWS0031 |  |
| EWS | AEWS1031 |  |
| EWS | AEWS0031 |  |
| EWS | AEWS1031 |  |
| EWS | AEWS1031, regimen A |  |
| EWS |  | vincristine, doxorubicin, cyclophosphamide / IE |
| EWS |  | Per AEWS0031, regimen B  ifosfamide, etoposide, cyclophosphamide, doxorubicin, vincristine |
| IFS | VDRIAC |  |
| RMS |  | Per ARST0331, subset B, regimen 2, VAC |
| RMS | ARST0531 |  |
| RMS | DD4A -> ICEx; cyclo/topo |  |
| RMS |  | Ifosfamide, carboplatin, etoposide |
| RMS | D9802; enrolled on D9902  Irinotecan, VCR, Cyclophosphamide |  |
| RMS |  | Per ARST0531:  Vincristine, Dactinomycin, Cyclophosphamide, and Irinotecan. |
| Synovial |  | Vincristine/Adriamycin/Ifosfamide |
| Synovial | ARST0332 |  |
| Undifferentiated | AREN0321 -> ARST0332 (Ifos/Doxo) |  |
| Undifferentiated |  | Per High-Risk Ewing protocol: ifosfamide, VP-16 |
| Undifferentiated | ARST0332, Arm C |  |
| Undifferentiated | AREN0321 -> ARST0332 |  |
| Undifferentiated |  | VAdriac, then cyclophosphamide, ifosfamide/doxorubicin |
| Undifferentiated |  | Cyclophosphamide, Dactinomycin, Irinotecan, and Vincristine. |
| Undifferentiated | ARST0531 |  |
| Undifferentiated |  | AEWS0031 protocol |
| Other Sarcoma | ARST0332, Arm C |  |
| Other Sarcoma | ARST0332 |  |
| Other Sarcoma |  | Per AREN0534 Regimen UH-3 -> AREN0533 Regimen DD-4A -> Regimen M; VDE/IE |
| Other Sarcoma |  | rapamycin |
| Other Sarcoma |  | Per AEWS1221: cyclophosphamide, doxorubicin, vincristine, etoposide |

*Each row represents an individual patient with histology shown in left column. Empty rows: no protocol or treatment plan documented; Chemotherapy: cyclophosphamide, etoposide, doxorubicin, vincristine, actinomycin, ifosfamide, temozolomide, topotecan, irinotecan. Undocumented protocol or regimen occurred with the following frequencies for each histology: CCSK, n=9; other sarcoma, n=5; infantile fibrosarcoma, n=2; RMS, n=1; synovial, n=1. CCSK: clear cell sarcoma of the kidney; EWS: Ewing sarcoma; RMS: rhabdomyosarcoma; IFS: infantile fibrosarcoma

**Supplemental Table 6. Health Outcomes and Survival**

|  | **N** | **CCSK**  **(N=94)** | **Ewing**  **(N=33)** | **Undifferent**  **(N=8)** | **Rhabdomyo**  **(N=7)** | **Infan Fibro**  **(N=3)** | **Synovial**  **(N=3)** | **Other**  **(N=10)** | **Combined**  **(N=158)** | **Statistic** |
| --- | --- | --- | --- | --- | --- | --- | --- | --- | --- | --- |
| **Dialysis** | 158 | 0.0% (0) | 6.1% (2) | 0.0% (0) | 0.0% (0) | 0.0% (0) | 0.0% (0) | 0.0% (0) | 1.3% (2) | P=0.26 |
| **Renal Transplant** | 158 | 0.0% (0) | 9.1% (3) | 0.0% (0) | 0.0% (0) | 0.0% (0) | 0.0% (0) | 0.0% (0) | 1.9% (3) | P=0.072 |
| **Hypertension** | 158 | 7.4% (7) | 15.2% (5) | 12.5% (1) | 0.0% (0) | 0.0% (0) | 0.0% (0) | 10.0% (1) | 8.9% (14) | P=0.78 |
| **Chronic Kidney Disease** | 158 | 11% (10) | 27% (9) | 25% (2) | 0% (0) | 33% (1) | 67% (2) | 20% (2) | 16% (26) | P=0.041 |
| **End-Stage Renal Disease** | 158 | 0.0% (0) | 9.1% (3) | 0.0% (0) | 0.0% (0) | 0.0% (0) | 0.0% (0) | 0.0% (0) | 1.9% (3) | P=0.072 |
| **Second Malignancy*** | 140 | 1.1% (1) | 9.1% (3) | 25.0% (2) | 14.3% (1) | 0.0% (0) | 0.0% (0) | 0.0% (0) | 5% (7) | P=0.024 |
| **No Long-term Complications** | 158 | 63% (59) | 33% (11) | 50% (4) | 29% (2) | 33% (1) | 0% (0) | 20% (2) | 50% (79) | P=0.006 |
| **Status Last Follow Up** | 145 |  |  |  |  |  |  |  |  | P=0.026 |
| Alive, NED |  | 87.4% (76) | 68.8% (22) | 85.7% (6) | 50.0% (3) | 66.7% (2) | 33.3% (1) | 71.4% (5) | 79.3% (115) |  |
| Alive with Disease  (in therapy) |  | 2.3% (2) | 6.2% (2) | 0.0% (0) | 0.0% (0) | 0.0% (0) | 33.3% (1) | 0.0% (0) | 3.4% (5) |  |
| Alive with Disease  (no therapy) |  | 3.4% (3) | 6.2% (2) | 0.0% (0) | 0.0% (0) | 0.0% (0) | 33.3% (1) | 14.3% (1) | 4.8% (7) |  |
| Deceased |  | 6.9% (6) | 18.8% (6) | 14.3% (1) | 50.0% (3) | 33.3% (1) | 0.0% (0) | 14.3% (1) | 12.4% (18) |  |
| **Cause of Death** | 18 |  |  |  |  |  |  |  |  | P=0.35 |
| Primary sarcoma |  | 67% (4) | 83% (5) | 100% (1) | 67% (2) | 0% (0) |  | 100% (1) | 72% (13) |  |
| Non-surgical therapy |  | 33% (2) | 0% (0) | 0% (0) | 0% (0) | 0% (0) |  | 0% (0) | 11% (2) |  |
| Unrelated |  | 0% (0) | 17% (1) | 0% (0) | 33% (1) | 100% (1) |  | 0% (0) | 17% (3) |  |
| **Survival Time** | 151 | 31 **66** 125  (86 +/- 64) | 28 **69** 115  (80 +/- 64) | 20 **120** 135  (83 +/- 64) | 31 **51** 71  (58 +/- 47) | 74 **112** 118 (91 +/- 48) | 31 **55** 70  (49 +/- 40) | 20 **42** 61  (58 +/- 66) | 29 **66** 124  (81 +/- 63) | *P=0.75 |

CCSK: clear cell sarcoma of the kidney. Ewing: Ewing sarcoma. Undifferent: undifferentiated sarcoma. Rhabdomyo: rhabdomyosarcoma. Infan Fibro: infantile fibrosarcoma. Other sarcomas include: “spindle cell neoplasms” or “renal sarcoma”, not otherwise specified (n=4), DICER1-associated anaplastic sarcoma (n=2), desmoplastic small round cell tumor (n=2), malignant perivascular epithelioid cell tumor (PEComa, n=1), and epithelioid sarcoma (n=1). Descriptive Statistics (N = 158). a **b** c represent the lower quartile, a, the median, **b**, and the upper quartile, c, for continuous variables. N is the number of non-missing values. Numbers after proportions are frequencies. Tests used: ^*^Kruskal-Wallis and Pearson tests (all others). Gray shade indicates no data. NED: no evidence of disease (i.e., primary sarcoma). *Second malignancy refers to a second malignant neoplasm distinct from the primary renal sarcoma and does not represent a relapse of or metachronous primary renal sarcoma.

**Supplemental Table 7: Survival Estimates**

1. **Survival Estimates by Histology**

**Overall Survival**

| **Histology Type** | **Survival Estimate** | **Lower 95% CI** | **Upper 95% CI** |
| --- | --- | --- | --- |
| CCSK | 0.9273 | 0.8449 | 0.9667 |
| EWS | 0.9008 | 0.7229 | 0.9669 |
| Undifferentiated | 0.833 | 0.273 | 0.975 |
| RMS | 0.500 | 0.111 | 0.804 |
| IFS | 0.667 | 0.0541 | 0.9452 |
| Other | 0.857 | 0.334 | 0.979 |

**Event-Free Survival**

| **Histology Type** | **Survival Estimate** | **Lower 95% CI** | **Upper 95% CI** |
| --- | --- | --- | --- |
| CCSK | 0.8890 | 0.7970 | 0.9409 |
| EWS | 0.9310 | 0.7514 | 0.9823 |
| Undifferentiated | 0.800 | 0.204 | 0.969 |
| RMS | 0.625 | 0.142 | 0.893 |
| Other | 0.752 | 0.407 | 0.914 |

1. **Survival Estimates by Stage**

**Overall Survival**

| **Stage** | **Survival Estimate** | **Lower 95% CI** | **Upper 95% CI** |
| --- | --- | --- | --- |
| Stage I | 1.0 | NA | NA |
| Stage II | 1.0 | NA | NA |
| Stage III | 0.9209 | 0.8021 | 0.9697 |
| Stage IV | 0.6044 | 0.3971 | 0.7600 |

**Event-Free Survival**

| **Stage** | **Survival Estimate** | **Lower 95% CI** | **Upper 95% CI** |
| --- | --- | --- | --- |
| Stage I | 0.8705 | 0.5733 | 0.9660 |
| Stage II | 0.9285 | 0.7943 | 0.9764 |
| Stage III | 0.9152 | 0.7890 | 0.9674 |
| Stage IV | 0.7327 | 0.5127 | 0.8652 |

1. **Survival Estimates by Metastatic Disease**

**Overall Survival**

| **Metastatic Disease** | **Survival Estimate** | **Lower 95% CI** | **Upper 95% CI** |
| --- | --- | --- | --- |
| YES | 0.7189 | 0.5481 | 0.8343 |
| NO | 0.9566 | 0.8879 | 0.9836 |

**Event-Free Survival**

| **Metastatic Disease** | **Survival Estimate** | **Lower 95% CI** | **Upper 95% CI** |
| --- | --- | --- | --- |
| YES | 0.8405 | 0.6760 | 0.9258 |
| NO | 0.8827 | 0.7979 | 0.9334 |

1. **Survival Estimates by Pulmonary Metastasis**

**Overall Survival**

| **Pulmonary Metastasis** | **Survival Estimate** | **Lower 95% CI** | **Upper 95% CI** |
| --- | --- | --- | --- |
| YES | 0.510 | 0.250 | 0.721 |
| NO | 0.9408 | 0.8794 | 0.9714 |

**Event-Free Survival**

| **Pulmonary Metastasis** | **Survival Estimate** | **Lower 95% CI** | **Upper 95% CI** |
| --- | --- | --- | --- |
| YES | 0.793 | 0.485 | 0.929 |
| NO | 0.8833 | 0.8104 | 0.9293 |

1. **Survival Estimates by Bone Metastasis**

**Overall Survival**

| **Bone Metastasis** | **Survival Estimate** | **Lower 95% CI** | **Upper 95% CI** |
| --- | --- | --- | --- |
| YES | 0.779 | 0.354 | 0.942 |
| NO | 0.8960 | 0.8272 | 0.9384 |

**Event-Free Survival**

| **Bone Metastasis** | **Survival Estimate** | **Lower 95% CI** | **Upper 95% CI** |
| --- | --- | --- | --- |
| YES | 0.714 | 0.337 | 0.901 |
| NO | 0.8873 | 0.8166 | 0.9318 |

1. **Survival Estimates by Molecular Alteration**

**Overall Survival**

| **Molecular Alteration** | **Survival Estimate** | **Lower 95% CI** | **Upper 95% CI** |
| --- | --- | --- | --- |
| YES | 0.9458 | 0.8412 | 0.9822 |
| NO | 0.8560 | 0.7600 | 0.9156 |

**Event-Free Survival**

| **Molecular Alteration** | **Survival Estimate** | **Lower 95% CI** | **Upper 95% CI** |
| --- | --- | --- | --- |
| YES | 0.8996 | 0.7740 | 0.9572 |
| NO | 0.8596 | 0.7612 | 0.9163 |

1. **Survival Estimates by Treatment Order**

**Overall Survival**

| **Treatment Order** | **Survival Estimate** | **Lower 95% CI** | **Upper 95% CI** |
| --- | --- | --- | --- |
| No surgery | 0.500 | 0.00598 | 0.91041 |
| Neoadjuvant then surgery | 0.9091 | 0.5081 | 0.9867 |
| Upfront surgery then adjuvant | 0.8911 | 0.8158 | 0.9367 |
| Neoadjuvant and adjuvant | 0.9091 | 0.5081 | 0.9867 |

**Event-Free Survival**

| **Treatment Order** | **Survival Estimate** | **Lower 95% CI** | **Upper 95% CI** |
| --- | --- | --- | --- |
| No surgery | 1.0 | NA | NA |
| Neoadjuvant then surgery | 0.711 | 0.394 | 0.833 |
| Upfront surgery then adjuvant | 0.8985 | 0.8237 | 0.9426 |
| Neoadjuvant and adjuvant | 0.787 | 0.381 | 0.943 |

1. **Survival Estimates by Radiation Therapy**

**Overall Survival**

| **Radiation Therapy** | **Survival Estimate** | **Lower 95% CI** | **Upper 95% CI** |
| --- | --- | --- | --- |
| YES | 0.9085 | 0.8312 | 0.9514 |
| NO | 0.8244 | 0.6480 | 0.9514 |

**Event-Free Survival**

| **Radiation Therapy** | **Survival Estimate** | **Lower 95% CI** | **Upper 95% CI** |
| --- | --- | --- | --- |
| YES | 0.859 | 0.774 | 0.914 |
| NO | 0.9151 | 0.7581 | 0.9720 |

**Supplemental Table 8. Cox regression**

Hazard Ratio and 95% CI; Clear cell sarcoma (CCSK) as reference histology

| Variable | HR | HR-low | HR-high | p-value |
| --- | --- | --- | --- | --- |
| **Overall Survival** |  |  |  |  |
| Age | 1.0 | 0.99 | 1.01 | 0.772 |
| Ewing | 3.50 | 0.64 | 19.09 | 0.148 |
| Undifferentiated | 2.65 | 0.30 | 23.35 | 0.379 |
| Rhabdomyosarcoma | 9.36 | 2.30 | 38.11 | 0.002 |
| Others* | 2.98 | 0.51 | 17.43 | 0.225 |
| **Event-free Survival** |  |  |  |  |
| Age | 1.0 | 0.99 | 1.01 | 0.677 |
| Ewing | 1.2 | 0.24 | 6.02 | 0.825 |
| Undifferentiated | 1.51 | 0.18 | 12.58 | 0.702 |
| Rhabdomyosarcoma | 5.25 | 1.12 | 24.53 | 0.035 |
| Others* | 2.21 | 0.45 | 10.95 | 0.330 |

*All histologies having three or less occurrences were combined as “Other” for Cox regression.
